# Fermented Soybean Meal Replacement in the Diet of Lactating Holstein Dairy Cows: Modulated Rumen Fermentation and Ruminal Microflora

**DOI:** 10.3389/fmicb.2021.625857

**Published:** 2021-01-29

**Authors:** Zuo Wang, Yuannian Yu, Xinyao Li, Hongyan Xiao, Peihua Zhang, Weijun Shen, Fachun Wan, Jianhua He, Shaoxun Tang, Zhiliang Tan, Duanqin Wu, Hui Yao

**Affiliations:** ^1^College of Animal Science and Technology, Hunan Agricultural University, Changsha, China; ^2^CAS Key Laboratory of Agro-Ecological Processes in Subtropical Region, National Engineering Laboratory for Pollution Control and Waste Utilization in Livestock and Poultry Production, Hunan Provincial Key Laboratory of Animal Nutrition & Physiology and Metabolism, Institute of Subtropical Agriculture, Chinese Academy of Sciences, Changsha, China; ^3^Institute of Bast Fiber Crops, Chinese Academy of Agricultural Sciences, Changsha, China; ^4^Nanshan Dairy Co., Ltd., Shaoyang, China

**Keywords:** dairy cow, fermented soybean meal, rumen fermentation, rumen microflora, third-generation sequencing

## Abstract

This study was conducted to examine the influences of replacing soybean meal (SBM) with fermented soybean meal (FSBM) in the diet of lactating Holstein cattle on rumen fermentation and ruminal bacterial microbiome. Twenty-four lactating Chinese Holstein dairy cattle were assigned to each of the two treatments in a completely randomized design: the SBM group [the basal total mixed ration (TMR) diet containing 5.77% SBM] and the FSBM group (the experimental TMR diet containing 5.55% FSBM). This trial lasted for 54 days (14 days for adjustment and 40 days for data and sample collection), and samples of rumen liquid were collected on 34 d and 54 d, respectively. The results showed that replacing SBM with FSBM significantly increased the molar percentages of propionate (*P* < 0.01) and valerate (*P* < 0.05), but reduced the total volatile fatty acid (TVFA) concentration (*P* < 0.05), butyrate molar proportion (*P* < 0.05), and the acetate to propionate ratio (*P* < 0.01). The copy numbers of total bacteria (*P* < 0.05), *Fibrobacter succinogenes* (*P* < 0.01), *Selenomonas ruminantium* (*P* < 0.01), and *Prevotella* spp. (*P* < 0.05) in the FSBM group were greater, while the density of *Prevotella ruminicola* (*P* < 0.05) was lower than those in the SBM treatment. Additionally, *Succiniclasticum ruminis* and *Saccharofermentans acetigenes* were significantly enriched (*P* < 0.05) in the rumen fluid of FSBM-fed cows, despite the fact that there was no remarkable difference in the Alpha diversity indexes, structure and KEGG pathway abundances of the bacterial community across the two treatments. It could hence be concluded that the substitution of FSBM for SBM modulated rumen fermentation and rumen bacterial microbiota in lactating Holstein dairy cows. Further research is required to elucidate the relevant mechanisms of FSBM, and provide more insights into the application of FSBM in dairy cattle.

## Introduction

As a costly and essential ingredient in the diet of dairy cattle, protein supplies ruminal microorganisms with amino acids and nitrogen for the synthesis of microbial protein, and meets the nutritional requirements of the host for various purposes (Liu et al., 2013; Imran et al., 2018). Soybean meal (SBM) is the most common and widely used protein source for the diets of lactating dairy cattle, and it possesses abundant rumen degradable protein (RDP), relatively balanced amino acid profile, and high digestibility of cellulose and pectin (Kwon et al., 2011; Imran et al., 2018). It has been verified that supplementing SBM resulted in the increments in dry matter intake, milk production, and milk protein concentration of grazing Holstein cows with *ad libitum* access to grass silage (Rego et al., 2008).

Nonetheless, SBM also has its own significant disadvantages, including low levels of rumen undegradable protein (RUP), low ratio of methionine (Met) to lysine (Lys), and the presence of multiple anti-nutritional agents (e.g., trypsin inhibitors, hemagglutinins, raffinose, and stachyose) (Yoo et al., 2009; Zhang et al., 2013; Imran et al., 2018). Fermentation could be an effective approach to promote the quality of SBM via microbial metabolism or microbial enzymatic activity (Chatterjee et al., 2018). Previous investigations have found that anti-nutritional factors might be eliminated or reduced through microbial fermentation of soybean meal (Feng et al., 2007; Wang et al., 2020a). Besides, it has been demonstrated that the fermentation process of soybean meal enhances the amount of non-protein nitrogen including small peptides, free amino acids, and ammonia (Feizi et al., 2020). Furthermore, the RUP content could also be elevated in fermented soybean meal (FSBM), possibly due to intensive heat processing before fermentation treatment (Stein et al., 2008).

To date, most studies on the application of FSBM in dairy cows have focused on calves. Kwon et al. (2011) reported that FSBM could help mitigate weaning stress and boost the immune function of weaned calves after experimentally induced lipopolysaccharide (LPS) challenge by increasing the levels of LPS-specific IgG, LPS-specific IgA, and haptoglobin, but reducing the cortisol concentration. It was further illustrated that apart from the alleviated weaning stress through the reduction of pro-inflammatory mediators, the growth performance was also enhanced by providing abruptly weaned Holstein calves with FSBM during cold weather (Rezazadeh et al., 2019). Subsequently, Feizi et al. (2020) concluded that substituting SBM with FSBM might promote the performance of calves by altering rumen fermentation products, as well as the relative abundance of specific ruminal bacteria. In contrast, information on the influences of dietary FSBM replacement in the lactating dairy cattle is rare.

The conversion of feed components into microbial mass and fermentation end products in ruminants is primarily dependent on the diverse microbiota (i.e., bacteria, archaea, protozoa, fungi and viruses) coexisting and interacting with each other in the complex ecosystem of the rumen (Wang et al., 2017a; McGovern et al., 2018). Consequently, the ruminal microbes and rumen microbial fermentation considerably affect the productivity, health, and wellbeing of the host (Yáñez-Ruiz et al., 2015; Wang et al., 2019). Henderson et al. (2015) found that the microbial community and fermentation function of the rumen are predominantly shaped by the diet. It is thus significant to elucidate the effects of FSBM application in the ration of lactating dairy cows on rumen fermentation and rumen microorganisms, especially with the aid of third-generation sequencing that targets the full-length 16S rRNA gene and offers higher accuracy and resolution of microbial communities than partial 16S rRNA gene sequencing (Klemetsen et al., 2019; He et al., 2020).

In this study, we aimed to explore the effects of replacing SBM with FSBM in the diet of lactating Holstein cattle on rumen fermentation and ruminal microbiota, to offer a better reference for the application of dietary FSBM in ruminants.

## Materials and Methods

This experiment was approved by the Animal Care Committee (approval number: 20190602), College of Animal Science and Technology, Hunan Agricultural University, Changsha, China.

### Animals, Diets, and Management

The present experiment was conducted at the Nanshan Dairy Farm (Shaoyang, Hunan Province, China). Twenty-four lactating Chinese Holstein dairy cows (initial mean ± SE; 20 ± 3.4 kg of milk/day, 164 ± 46 days in milk, 2 ± 1 of parity, and 460 ± 50 kg of body weight) were used as the experimental animals in this trial. Cattle were allocated to each of the two treatments comprising the SBM group (the basal TMR diet containing 5.77% SBM) and the FSBM group (the test TMR diet containing 5.55% FSBM), in a completely randomized design. The FSBM used in this trial was a commercial product and fermented with the inoculation of *Lactobacillus* spp., *Bacillus subtilis*, and *Saccharomyces cerevisae* (Minxiong Biotechnology Co., Ltd., Longyan, China). The components and chemical compositions of the two rations are displayed in Table 1. This experiment lasted for 54 days, consisting of 14 days for adjustment and 40 days for data and sample collection. All cows were housed in a tie stall barn, and fed *ad libitum* twice per day at 06:00 h and 18:00 h with free access to fresh water.

**TABLE 1:** Ingredients and nutrient contents of diets for the SBM group and FSBM group.

	**SBM^1^**	**FSBM^2^**

**Ingredients,% DM**		
Corn	17.68	17.89
Wheat flour	4.7	4.7
Corn germ meal	1.75	2.44
DDGS^3^	6.41	5.77
Sprayed corn bran	5.13	5.13
Soybean meal	5.77	–
Fermented soybean meal	–	5.55
Alfalfa grass	8.62	8.62
Oat grass	4.42	4.42
*Leymus chinensis* hay	4.44	4.44
Corn silage	33.36	33.36
Whole cottonseed	5.95	5.95
Urea	0.04	–
NaCl	0.27	0.27
CaHCO_3_	0.44	0.44
CaCO_3_	0.56	0.56
Premix^4^	0.46	0.46

**Nutrient contents,% DM**		

NE_L,_ Mcal/kg	1.61	1.61
Organic matter	91.50	91.40
Crude protein	15.87	15.92
Neutral detergent fiber	35.50	36.00
Acid detergent fiber	22.81	22.95
Ether extract	4.33	4.33
Ash	8.50	8.60
Ca	0.51	0.52
P	0.39	0.39

*^1^SBM, soybean meal; ^2^FSBM, fermented soybean meal; ^3^DDGS, distillers’ dried grains with soluble; ^4^Every 1 kg of premix contained 400 mg of Zn, 100 mg of Cu, 200 mg of Fe, 3,600 mg of Mg, 350 mg of Mu, 96 mg of Cr, 4.0 mg of Co, 50 mg of Se, 500 mg of lysine, 500 mg of methionine, 25,00,000 IU of vitamin A, 1,00,000 IU of vitamin D3, and 4,000 IU of vitamin E.*

### Sampling

The sampling was performed on 34 and 54 d of the experimental period, respectively. On each sampling day, rumen fluid from the central rumen of each cow was respectively collected 2 h before and 4 h after morning feeding through the oral cavity using an oral stomach tube as described by Shen et al. (2012). In brief, the first 150 mL of rumen liquid was discarded, and then another 150 mL was obtained and further strained through four layers of cheesecloth under a continuous CO_2_ stream. For measuring rumen fermentation characteristics, the rumen liquid samples were collected from all the dairy cattle at each time point of each sampling day. As to the DNA extraction and relevant analysis, six cows from each treatment were firstly randomly selected. On each sampling day, the rumen fluid samples collected from those six cows of each treatment at the two time points were mixed together at the ratio of 1: 1. All samples were immediately frozen in liquid nitrogen and then stored at −80°C until further analysis.

### Chemical Analysis

The dry matter (DM; method 930.15), ash (method 942.05), crude protein (method 2001.11), ether extract (method 920.39), neutral detergent fiber (NDF; method 2002.04), and acid detergent fiber (ADF; method 973.18) of the two diets were analyzed according to the procedures of AOAC (2005). The contents of calcium (Ca) and phosphorus (P) in the two rations were measured as previously depicted (Tang et al., 2019; Wang et al., 2020b).

The measurement for the pH of rumen fluid was performed with a pH meter (PHS-3C, INESA Scientific Instrument Co., Ltd., Shanghai, China) immediately after sampling. The concentrations of ammonia nitrogen (NH_3_-N) and volatile fatty acid (VFA) were determined employing the methods described by Wang et al. (2016b). Briefly, in calculating the NH_3_-N concentration, a spectrophotometer (UV-2300; Shimadzu, Kyoto, Japan) was used to detect the light absorption value at 700 nm. The VFA analysis was performed using a DB-FFAP gas chromatograph (HP5890, Agilent Technologies, Palo Alto, United States).

### DNA Extraction and Real-Time Quantitative PCR

The isolation of genomic DNA from the rumen fluid was conducted with a phenol-free bead-beating method reported by Yu and Morrison (2004). The quality and quantity of extracted DNA were measured on a ND-1000 spectrophotometer (NanoDrop Technologies Inc., Wilmington, United States). Absolute real-time quantitative PCR (RT-qPCR) was adopted to determine the copy numbers of the 16S/18S rRNA genes of total bacteria, anaerobic fungi, methanogenic archaea, *Prevotella* spp., *Prevotella ruminicola*, *Ruminococcus flavefaciens*, *Fibrobacter succinogenes*, *Selenomonas ruminantium*, and *Ruminobacter amylophilus* in rumen fluid. The specific primers for each targeted microorganism in this experiment have been validated in previous studies (Koike et al., 2003; Denman and McSweeney, 2006; Stevenson and Weimer, 2007; Hook et al., 2009). RT-qPCR was performed on a 96-well ABI 7900HT (Applied Biosystems, Foster City, United States) with a total 10 μL-reaction mix, using DyNAmo HS SYBR Green qPCR 2 × master mix (Thermo Fisher Scientific, Waltham, United States) as described antecedently (Wang et al., 2017b). A standard curve was generated for each targeted microbe using plasmid DNA containing the exact 16S or 18S rRNA gene inserts. The linear relationship observed between the threshold amplification (C_t_) and the logarithm of 16S or 18S rRNA copy numbers of the standards was used to calculate the copy numbers of targeted microorganisms per mL of rumen fluid. Each estimate was a mean of triplicates.

### PCR Amplification and Full-Length 16S rRNA Gene Sequencing

For the full-length 16S rRNA gene sequencing, the rumen liquid samples from six dairy cows of each treatment were randomly selected. The full-length bacterial 16S rRNA genes were firstly amplified based on the genomic DNA isolated from rumen fluid using universal primers 27F (5′-AGRG TTTGATYNTGGCTCAG-3′) and 1492R (5′-TASGGHTACCT TGTTASGACTT-3′) with barcode in accordance with a precedent study (Klemetsen et al., 2019). All PCR reactions were performed in a 10 μL reaction containing 100 ng of extracted template DNA, 0.3 μL of each forward and reverse primers (10 μM), 2 μL of dNTP (2 mM each), 5 μL of KOD FX Neo Buf (Toyobo Co., Ltd., Osaka, Japan), and 0.2 μL of KOD FX Neo. The thermal cycling procedures were as follows: initial denaturation at 95°C for 5 min; 30 cycles of denaturation (95°C, 30 s), annealing (50°C, 30 s), and elongation (72°C, 1 min); and a final extension at 72°C for 7 min. Each DNA sample was amplified in duplicates, and three wells per run served as the negative control. Duplicate PCR products were mixed, and the correct sizes of PCR products and the absence of signal from negative controls were further verified with agarose gel electrophoresis. The barcoded 16S rRNA gene amplicons were quantified using the Qubit 2.0 Fluorometer (Thermo Fisher Scientific, Waltham, United States). The amplicons were then pooled in equimolar concentrations and purified with the PureLink PCR Purification Kit (Thermo Fisher Scientific, Waltham, United States). The amplicon sequencing library was constructed using PacBio 2 kb library preparation protocol (Pacific Biosciences, Menlo Park, United States). After the library QC was finished, the qualified library was sequenced on a PacBio Sequel II platform (Pacific Biosciences, Menlo Park, United States) and single-end reads were generated.

### Bioinformatic Analysis

The bioinformatics analysis of this study was performed with the aid of the BMK Cloud (Biomarker Technologies Co., Ltd., Beijing, China). The raw reads generated from sequencing were filtered and demultiplexed using the SMRT Link software (version 8.0) with the *minPasses* ≥5 and *minPredictedAccuracy* ≥0.9, in order to obtain the circular consensus sequencing (CCS) reads. Subsequently, the lima (version 1.7.0)^1^ was employed to assign the CCS sequences to the corresponding samples based on their barcodes. CCS reads containing no primers and those reads beyond the length range (1,200–1,650 bp) were discarded through the recognition of forward and reverse primers and quality filtering using the Cutadapt quality control process (version 2.7)^2^. The UCHIME algorithm (Edgar et al., 2011) (V8.1)^3^ was used in detecting and removing chimera sequences to obtain the clean reads. Sequences with similarity ≥97% were clustered into the same operational taxonomic unit (OTU) by USEARCH (V10.0), and the OTUs with reabundace <0.005% were filtered (Quast et al., 2012). Taxonomy annotation of the OTUs was performed based on the RDP Classifier (version 2.2)^4^ (Wang et al., 2007) using the SILVA database (release132)^5^ with a confidence threshold of 80%. To enable calculation of Unifrac distances (Lozupone et al., 2011) and to facilitate downstream diversity analysis, the picked OTUs were aligned by PyNAST (V1.2.2)^6^ (Caporaso et al., 2010) against the core alignment template of SILVA database, and a phylogenetic tree was constructed using MEGAN5^7^ (Huson et al., 2007). The OTUs abundance information was normalized using a standard of sequence number corresponding to the sample with the fewest sequences, and further analysis on the Alpha diversity and Beta diversity were carried out based on the normalized output data. All the sequences in the current study were deposited to the sequence read archive (SRA) of the NCBI database under the accession number SRP274245.

The Alpha diversity indices were calculated and displayed by the QIIME (V1.8.0) and R software (V3.1), respectively. Beta diversity was computed employing the weighted and unweighted UniFrac distance matrix via QIIME (V1.8.0), and visualized with principal coordinate analysis (PCoA) plots displayed by the WGCNA package, stat packages and ggplot2 package in R software (V3.1). The function prediction via Tax4Fun (Aßhauer et al., 2015) was achieved by extracting the prokaryotic whole genome 16S rRNA gene sequence of KEGG database and aligning it to the SILVA SSU Ref NR database based on the minimum 16S rRNA sequence similarity to establish a correlation matrix, using the BLASTN algorithm (BLAST Bitscore >1,500). Further, the prokaryotic whole genome functional information of the KEGG database annotated by UProC, and PAUDA was mapped to the SILVA database to implement the SILVA database function annotation. The sequenced samples were clustered out of the OTU using the SILVA database sequence as a reference sequence.

### Statistical Analysis

To assess the effect of replacing SBM with FSBM in the diet of lactating dairy cows, data of the rumen fermentation characteristics in this study were analyzed using the PROC MIXED procedure of SAS (ver. 9.4, SAS Institute Inc.). The statistical model included treatment, sampling date, and the interaction between treatment and sampling time point as the fixed effects, with sampling date as the repeated measurement and animal as the random effect. Analysis of the data of ruminal microbial copy numbers and bacterial Alpha diversity indices was conducted with the PROC MIXED procedure with treatment and sampling date as the fixed effects, sampling date as repeated determination, and animal as the random effect. Least squares means are reported throughout the text. Statistical difference was respectively declared as significant or highly significant at *P* < 0.05 or *P* < 0.01, while trend was discussed at 0.05 < *P* ≤ 0.10. Linear discriminant analysis effect size (LEfSe) was used to compare relative abundances of microbial taxa between the two treatment groups, and significant differences were considered by a linear discriminant analysis (LDA) score >3 and *P* < 0.05.

## Results

### Rumen Fermentation Characteristics in Response to FSBM Replacement

In the present trial, the TVFA concentration (*P* < 0.01) and the molar percentages of butyrate (*P* < 0.01) and valerate (*P* < 0.05) were significantly affected by the sampling time point, while no significant (*P* > 0.05) interaction between treatment and sampling time point was observed (Table 2). Replacing SBM with FSBM significantly reduced the TVFA concentration (*P* < 0.05), butyrate molar percentage (*P* < 0.05), and the ratio of acetate to propionate (*P* < 0.01). By contrast, the molar percentages of propionate (*P* < 0.01) and valerate (*P* < 0.05) were both elevated significantly in response to the FSBM replacement. It was also noteworthy that the substitution of FSBM for SBM tended to lower (*P* < 0.1) the molar ratio of acetate in the rumen fluid.

**TABLE 2:** Comparison of rumen fermentation characteristics between the SBM group and FSBM group.

**Item**	**Treatment**		**SEM^3^**	***P*-Value**		
	**SBM^1^**	**FSBM^2^**		**Tr^4^**	**Tm^5^**	**Tr × Tm^6^**
pH	6.38	6.42	0.041	0.579	0.360	0.976
NH_3_-N (mmol/L)	7.00	6.51	0.40	0.366	0.168	0.876
TVFA^7^ (mmol/L)	96.5^a^	89.3^b^	2.21	0.020	0.001	0.432
**VFA profile (mol/100 mol)**						
Acetate	65.0	64.3	0.29	0.064	0.105	0.233
Propionate	18.9^b^	20.0^a^	0.21	0.001	0.725	0.938
Isobutyrate	0.92	0.95	0.026	0.371	0.168	0.313
Butyrate	12.3^a^	11.9^b^	0.14	0.046	0.002	0.127
Isovalerate	1.48	1.42	0.032	0.192	0.446	0.170
Valerate	1.40^b^	1.50^a^	0.030	0.014	0.018	0.071
A:P^8^	3.45^a^	3.26^b^	0.047	0.003	0.817	0.852

*^*a,b*^Means within a row for treatments that do not have a common superscript differ. ^1^SBM, soybean meal; ^2^FSBM, fermented soybean meal; ^3^SEM for treatment × sampling time point; ^4^Tr, treatment; ^5^Tm, sampling time point; ^6^Interaction between treatment and sampling time point; ^7^TVFA, total volatile fatty acid; ^8^A:P, ratio of acetate to propionate.*

### Copy Numbers of Target Ruminal Microbes in Response to FSBM Replacement

In the FSBM treatment, the copy numbers of total bacteria (*P* < 0.05), *F. succinogenes* (*P* < 0.01), *Selenomonas ruminantium* (*P* < 0.01), and *Prevotella* spp. (*P* < 0.05) were significantly higher, whist the *P. ruminicola* copy number was significantly less (*P* < 0.05) compared with those in the SBM group (Table 3). Replacing SBM with FSBM did not exert significant (*P* > 0.05) influences on the quantities of the remaining targeted microbes in this study.

**TABLE 3:** Comparison of copy numbers (Log_10_ copies / mL) of target microbes in the rumen fluid between the SBM group and FSBM group.

**Microbe**	**Treatment**		**SEM^3^**	***P*-Value**
	**SBM^1^**	**FSBM^2^**		
Total bacteria	12.60^b^	12.74^a^	0.043	0.025
*Ruminococcus flavefaciens*	8.84	9.12	0.126	0.105
*Fibrobacter succinogenes*	10.78^b^	11.20^a^	0.078	0.001
*Selenomonas ruminantium*	10.30^b^	10.53^a^	0.052	0.002
*Ruminobacter amylophilus*	9.01	9.18	0.191	0.433
*Prevotella* spp.	12.36^b^	12.53^a^	0.052	0.022
*Prevotella ruminicola*	12.03^a^	11.83^b^	0.121	0.038
Fungi	9.00	8.89	0.094	0.396
Methanogens	9.71	9.84	0.191	0.433

*^*a,b*^Means within a row for treatments that do not have a common superscript differ. ^1^SBM, soybean meal; ^2^FSBM, fermented soybean meal; ^3^SEM for treatments.*

### Taxonomic Identification of Rumen Bacteria Across Treatments

In the present study, the full-length 16S rRNA gene sequencing altogether generated 1,25,664 CCS sequences throughout all the rumen liquid samples with an average of 5227 ± 801 CCS sequences after filtering, and the average OTU number per sample was 277 ± 32 (Supplementary Table S1). The rarefaction curves on the number of OTUs indicated that the sequencing depth in this experiment was sufficient to characterize the bacterial microflora in the rumen fluid samples (Supplementary Figure S1). A total of 17 bacterial phyla were observed amongst all the samples, and *Firmicutes* (46.1 ± 8.74%), *Bacteroidetes* (30.4 ± 6.31%), and *Proteobacteria* (7.6 ± 6.43%) were the most predominant phyla, whilst the phyla *Verrucomicrobiota* (3.1 ± 2.29%) and *Planctomycetota* (2.5 ± 1.34%) were less abundant (Supplementary Figure S2). At the genus level, 108 bacterial genera were observed across all the samples. The three most dominant genera were successively *Prevotella* (23.0 ± 5.60%), *Succiniclasticum* (18.0 ± 10.08%), and *Ruminococcus* (1.9 ± 1.47%) (Supplementary Figure S3). In all, 130 bacterial species were found from all the rumen fluid samples of this study, and the bacterial communities across samples were primarily predominated by *Succiniclasticum ruminis* (18.0 ± 10.08%), *P. ruminicola* (4.3 ± 1.23%), and *Gabonia massiliensis* (1.4 ± 0.78%) (Supplementary Figure S4). In total, 521 and 520 OTUs were clustered in the SBM and FSBM group, in which 8 and 7 were exclusive to each treatment, respectively. More specifically, 7 of the unique OTUs in the SBM treatment were assigned to the phylum *Firmicutes*, while only 1 of them were annotated as the member of the phylum *Verrucomicrobiota* (Supplementary Table S2). In contrast, 4 of the exclusive OTUs in the FSBM group were identified as belonging to the phylum *Bacteroidetes*, with another OTU assigned to the phylum *Firmicutes* and the remaining 2 OTUs unclassified.

### Diversity of Rumen Bacterial Microbiota in Response to FSBM Replacement

**FIGURE 1:**
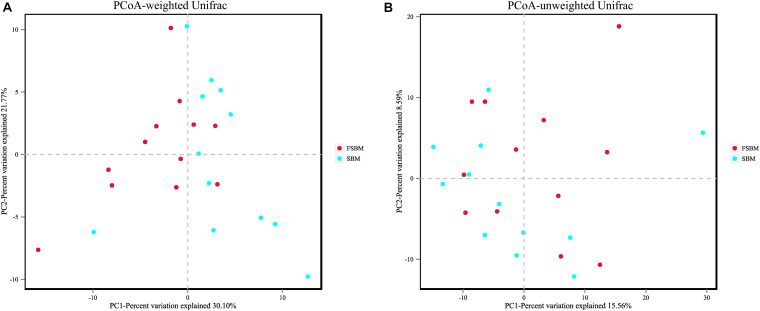
Principal coordinate analysis (PCoA) of bacterial community structure between the SBM and FSBM treatments. **(A)** PCoA based on weighted Unifrac matrix. **(B)** PCoA based on unweighted Unifrac matrix.

For the Alpha diversity, the indexes of ACE, Chao1, Shannon, and Simpson were adopted to measure and compare the bacterial diversity within the two groups (Table 4). All these four indexes were unaffected (*P* > 0.05) by replacing SBM with FSBM in the diet of dairy cows. As for the Beta diversity of the bacterial communities in rumen fluid between two treatments, the PCoA analysis was conducted and illustrated based on both weighted and unweighted Unifrac distances (Figure 1). The clustering of bacterial microbiota from the two treatments overlapped, and hence no clear distinction was noticed.

**TABLE 4:** Comparison of bacterial Alpha diversity indices between the SBM group and FSBM group.

**Alpha diversity index**	**Treatment**		**SEM^3^**	***P*-Value**
	**SBM^1^**	**FSBM^2^**		
ACE	420	419	16.9	0.963
Chao 1	392	408	11.6	0.328
Shannon	4.49	4.81	0.128	0.082
Simpson	0.05	0.03	0.012	0.144

*^1^SBM, soybean meal; ^2^FSBM, fermented soybean meal; ^3^SEM for treatments.*

### Differential Rumen Bacterial Taxa Between SBM and FSBM Treatments

**FIGURE 2:**
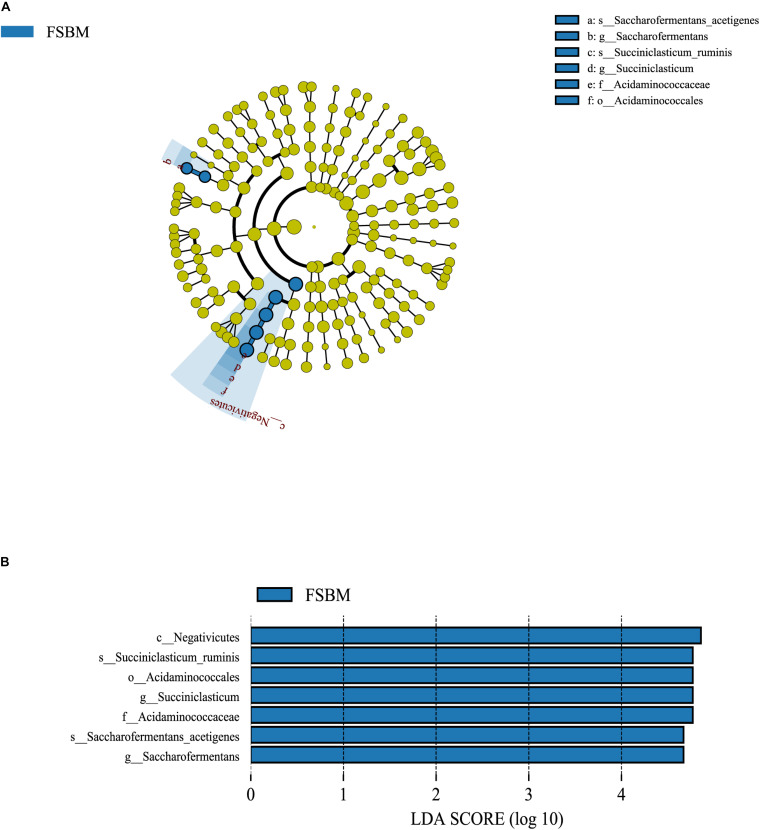
The LDA effect size (LEfSe) analysis of bacterial taxa between the SBM and FSBM treatments. **(A)** Cladogram displays significantly enriched bacterial taxa (from the class to the species level). Green: taxa abundant in the FSBM treatment. **(B)** Bar chart displays LDA scores of the SBM and FSBM treatments. The LDA scores represented the difference in relative abundance with exponent fold change of 10 between two treatments. Significant differences are defined as *P* < 0.05 and LDA score >3.0.

It was demonstrated through the LEfSe analysis that the most differentially abundant bacterial taxa at the genus level in the FSBM treatment were assigned to *Succiniclasticum* spp. and *Saccharofermentans* spp. (Figure 2). Moreover, *Succiniclasticum ruminis* and *Saccharofermentans acetigenes* were the most differentially abundant species in the FSBM treatment. All the differential bacterial taxa in the FSBM treatment weighted at similar degrees to the difference between the two groups, with an absolute LDA score >4.

### Function Prediction of Rumen Bacteria Across Treatments

**FIGURE 3:**
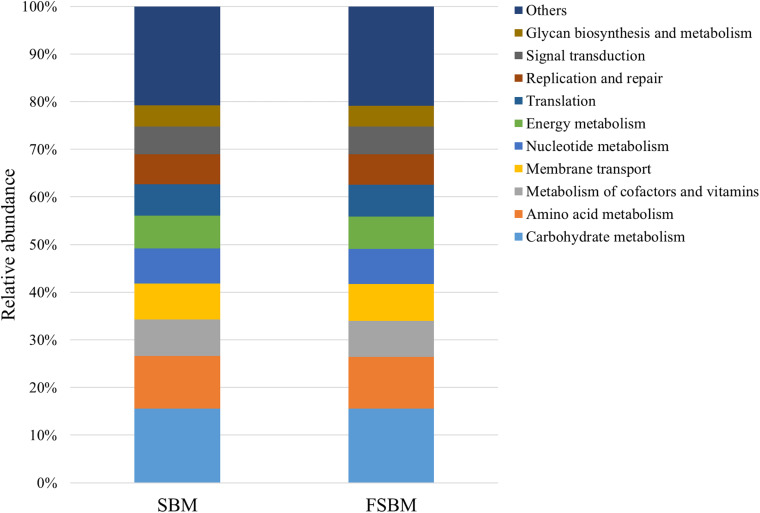
The top ten annotated KEGG pathways (at level 2) across the two treatments based on Tax4Fun function prediction.

**FIGURE 4:**
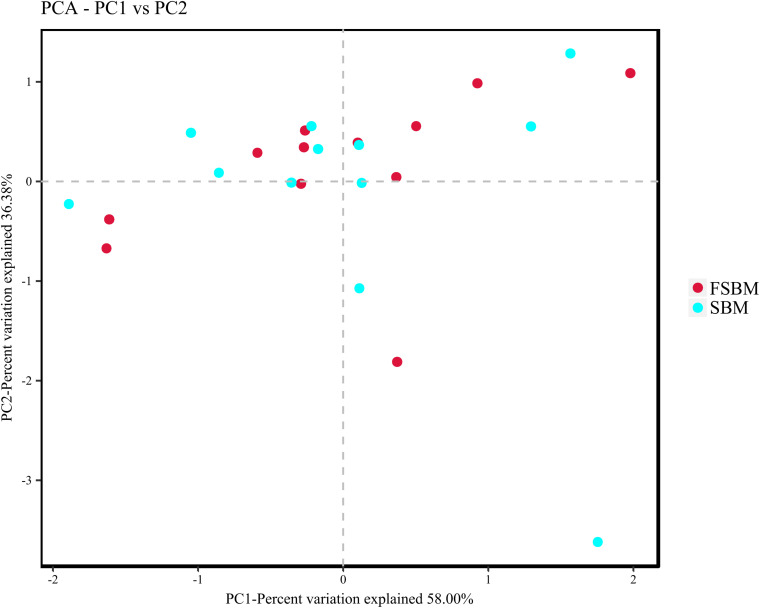
Principle component analysis (PCA) plotting for the predicted metagenome between the SBM and FSBM treatments based on Tax4Fun function prediction.

During the process of function prediction using Tax4Fun, 22.6 (±3.27)% of OTUs mapped to the SILVA database were allocated to KEEG orthologs (KO) and relevant pathways (at level 2). Amongst the top 10 assigned KEGG pathways across the SBM and FSBM groups, the KO abundances were primarily predominated by the carbohydrate metabolism, amino acid metabolism, metabolism of cofactors and vitamins, and membrane transport (Figure 3). As was depicted in the principle component analysis (PCA) chart, no explicit discrimination between the assigned KEGG pathways of two treatments was observed (Figure 4). In addition, based on the *t*-test, there was no significant (*P* > 0.05) discrepancy in the KO abundances of the overwhelming majority of the annotated pathways between the SBM and FSBM treatments (Supplementary Table S3).

## Discussion

In contrast to the SBM, it is acknowledged that FSBM is more preferred as a protein source for domestic ruminants since it could be characterized by the less amount of anti-nutritional agents, greater content of non-protein nitrogen (i.e., small peptides, free amino acids, and ammonia), and increased quantity of RUP (Zhang et al., 2013; Feizi et al., 2020; Wang et al., 2020a). Nonetheless, so far, most of the preceding investigations on the impacts of applying FSBM in dairy cattle have concentrated on the growth performance and immune response of young calves, while few have explored the effects of FSBM on the rumen fermentation and the ruminal bacterial microflora of lactating dairy cows (Kim et al., 2010, 2012; Kwon et al., 2011; Rezazadeh et al., 2019). In the current study, results showed that entirely replacing SBM with FSBM in the diet for lactating Holstein dairy cows did not affect the ruminal pH or the NH_3_-N concentration. However, Feizi et al. (2020) concluded that the substitution of FSBM for SBM at 50% in the starter diet raised the level of NH_3_-N in the rumen fluid of Holstein calves in a previous study, in which the ruminal pH was not measured. This inconsistency can be attributed to the differences in the animals, composition of experimental rations, and contents of FSBMs, and further research is required.

As was revealed in the results of the current trial, replacing SBM with FSBM significantly decreased the TVFA concentration and the molar ratio of butyrate, while tended to reduce the acetate molar proportion. Similarly, Feizi et al. (2020) found that the substitution of FSBM for SBM at 30 and 50% both significantly lowered the molar ratio of acetate in the rumen fluid of Holstein calves, while the molar proportion of butyrate tended to be less in the calves fed the starter in which SBM was replaced by FSBM at 30%. This phenomenon could be explained by the greater quantity of RUP in FSBM (Stein et al., 2008; Rezazadeh et al., 2019), as it has been reported that the TVFA production and the molar percentages of acetate and butyrate was lessened in response to the existence of RUP in a dual-flow continuous culture system using rumen contents obtained from Holstein cattle (Griswold et al., 1996). In addition, it was noticeable that the valerate molar proportion was elevated as SBM was replaced by FSBM in this study, which is consistent with the results of Feizi et al. (2020). The increment in the valerate production could possibly result from the higher content of small peptides in FSBM compared to SBM, since the valeric acid principally originates from the ruminal degradation of dietary proteins including small peptides (Allison and Bryant, 1963).

According to the RT-qPCR assay of the present study, it was observed that the copy number of total bacteria was increased in response to the FSBM replacement, and this result might partially be ascribed to the surge in the densities of *F. succinogenes*, *Selenomonas ruminantium*, and *Prevotella* spp., all of which were amongst the most abundant bacteria of the targeted microorganisms in this experiment. As a main propionate-producer present in the rumen ecosystem, *Selenomonas ruminantium* has been confirmed to utilize lactate to generate propionate (Hobson et al., 1963; Flythe and Aiken, 2010; Sawanon et al., 2011). Therefore, the higher density of *Selenomonas ruminantium* in the rumen fluid of FSBM-fed cows could be an explanation for the augment in the propionate molar ratio when SBM was replaced by FSBM in this study. Besides, the greater copy number of *Prevotella* taxon in the FSBM treatment compared to the SBM treatment might be induced by the increased amount of small peptides and/or amino acids in FSBM than SBM (Kim et al., 2012), as the *Prevotella* spp. have been found to be a member of the eminent proteolytic bacteria inside the rumen (Griswold, 1999; Sales-Duval et al., 2002). Nevertheless, it was also marked that the density of *P. ruminicola* was lowered by the FSBM replacement. Since *P. ruminicola* is not the sole species belonging to the genus *Prevotella*, hence it could be speculated that the increasing densities of other species (e.g., *P. bryantii* and *P. brevis*) might lead to the overall growth in the copy number of *Prevotella* spp. (Griswold, 1999). Furthermore, Feizi et al. (2020) found an increment in the *P. ruminicola* account in response to the substitution of FSBM for SBM at 50%. This contradiction could also probably result from the discrepancies in the hosts, contents of the diets, and components of FSBMs between that previous investigation and the present experiment.

Many studies, including our previous investigations, have verified that *Bacteroidetes*, *Firmicutes*, and *Proteobacteria* are primarily the three most predominant bacterial phyla existing within the rumen ecosystem despite different ages or regions of the ruminants, employing the 16S rRNA amplicon sequencing based on either DNA or RNA isolated from the rumen contents (Jami et al., 2013; Rey et al., 2014; Liu et al., 2016; Wang et al., 2016a, 2017a, 2020c). The current trial also showed that the ruminal microbiota at the phylum level was sequentially dominated by *Firmicutes*, *Bacteroidetes*, and *Proteobacteria* via the full-length 16S rRNA gene sequencing, which further confirmed the conclusions of previous studies. At the genus level, the successive dominance of the genera *Prevotella*, *Succiniclasticum*, and *Ruminococcus* was observed in this study. The ruminal *Prevotella* spp. have been reported to generally ferment hemicellulose, starch, protein, peptides, and pectin into succinate, propionate, and acetate (Carberry et al., 2012; Wang et al., 2019), while the genus *Succiniclasticum* is a constituent among the core rumen microflora, possessing the capability to convert succinate into propionate (Hook et al., 2011; Petri and Vahmani, 2018). Besides, the *Ruminococcus* spp. are the crucial cellulolytic bacteria commonly detected in the rumen (Flint and Bayer, 2008). In this study, the taxonomic profiling of the rumen bacterial community at the species level revealed the predominance of *Succiniclasticum ruminis*, *P. ruminicola*, and *Gabonia massiliensis*. The former two bacteria were the typical members of the genera *Succiniclasticum* and *Prevotella*, respectively. As an anaerobic Gram-negative and catalase-positive bacterium (Mourembou et al., 2016), the role of *Gabonia massiliensis* during rumen fermentation is currently indefinite, necessitating further investigations.

The diversity and richness of the rumen microbiome are essential factors impacting the functioning of rumen (Weimer, 2015; Xie et al., 2018). As was illustrated in the results of the present study, all of the indexes for Alpha diversity of the rumen bacterial microflora were unaffected by the FSBM replacement in the ration of lactating Holstein cows. Moreover, the PCoA plotting based on weighted and unweighted Unifrac distances both depicted that substituting FSBM for SBM did not reshape the structure of the bacterial microbiota within the rumen liquid of dairy cattle. The disparity between this phenomenon and the altered rumen fermentation parameters above in response to the replacement of SBM by FSBM might be to some extent explained by the relatively small differences in VFA profiles within treatments, and the underlying reason needs to be clarified in future studies.

The relative abundances of microbial taxa across the SBM and FSBM treatments in this trial were contrasted using the LEfSe analysis with the threshold for the LDA score set at 4, which revealed that *Succiniclasticum ruminis* and *Saccharofermentans acetigenes* are the two bacterial species significantly enriched in the rumen fluid of cattle of FSBM group. *Succiniclasticum ruminis* is a ruminal anaerobic and Gram-negative bacterium that ferments succinate to propionate as the single energy-producing mechanism (van Gylswyk, 1995), hence its enhancement in the FSBM group could be a logical explanation for the augment in the propionate molar proportion in the rumen liquid of the FSBM-fed cattle. Further, as introduced above in the results of RT-qPCR, replacing SBM with FSBM elevated the copy number of *F. succinogenes*, which has been proved to generate succinate as a primary output of carbohydrate degradation and hence provide *Succiniclasticum ruminis* with succinate as the substrate of fermentation (Scheifinger and Wolin, 1973). The consistent enrichments of these two bacteria in the present study again validated their mutualistic interactions involving the production of succinate and propionate during the rumen fermentation. As for the *Saccharofermentans acetigenes*, it has been reported by Chen et al. (2010) as an anaerobic Gram-negative bacterium that converts glucose mainly into acetate, lactate, and fumarate. Since the molar percentage of acetate in the rumen fluid of FSM-fed cows was decreased, the production of lactate and/or fumarate might be enhanced due to the enrichment of *Saccharofermentans acetigenes* in the FSBM treatment. However, this hypothesis requires future studies to be examined.

According to the Tax4Fun analysis of the current study, no remarkable difference in the abundances of KEGG pathways was shown through both the PCA plotting and the t-test, which might be inconsistent to the altered rumen fermentation characteristics exhibited above. The relatively small differences in the VFA profiles across treatments in this trial might be a possible cause of this discrepancy. In addition, the drawbacks of Tax4Fun mainly in functional databases and phylogenetic trees as declared previously (Iwai et al., 2016), could also be taken into account. Moreover, the fact that the solid-phase bacteria takes up the majority of the total ruminal bacterial populations, and the distinction between the bacterial microbiota in the liquid and solid fractions might be a possible cause collectively (Wang et al., 2017a). It should also be understood that the meta-genomic analysis might not always accurately reveal the factual metabolisms of the disparate and intricate microbiome inside the rumen ecosystem, as discussed in precedent studies (Lam et al., 2015; Ma et al., 2016; Morton et al., 2019).

## Conclusion

This study, as far as we are concerned, was the initial exploration on the effects of substituting FSBM for SBM on the rumen bacterial microbiota as well as the rumen fermentation parameters in lactating dairy cattle, by the aid of third-generation full-length 16S rRNA gene sequencing. It was noticed that, in response to the FSBM replacement, the molar proportions of propionate and valerate were increased, whilst the TVFA concentration, the butyrate molar percentage, and the ratio of acetate to propionate were lowered. Furthermore, despite the reduction in the copy number of *P. ruminicola*, the densities of the total bacteria, *F. succinogenes*, *Selenomonas ruminantium*, and *Prevotella* spp. in the rumen fluid were raised by replacing SBM with FSBM. Moreover, the substitution of FSBM for SBM enriched the bacterial species of *Succiniclasticum ruminis* and *Saccharofermentans acetigenes*. The current trial elucidated the variations in rumen fermentation and rumen bacterial microbiome induced by FSBM replacement in the ration for lactating dairy cows. To uncover the mechanisms in the modulations of FSBM on rumen fermentation and rumen microflora and expand references for the application of FSBM in the ruminants industry, deeper investigations need to be conducted in the future.

## Data Availability Statement

The datasets presented in this study can be found in online repositories. The names of the repository/repositories and accession number(s) can be found in the article/Supplementary Material.

## Ethics Statement

The animal study was reviewed and approved by the Animal Care Committee, College of Animal Science and Technology, Hunan Agricultural University.

## Author Contributions

ZW, ST, and ZT designed the research. ZW, YY, XL, HX, PZ, WS, FW, JH, HY, DW, and ST conducted the research. ZW, YY, and ST analyzed the data. ZW, DW, and ST wrote the manuscript. All authors approved the final manuscript.

## Conflict of Interest

HY was a member of the Nanshan Dairy Company. The remaining authors declare that this study was carried out without any commercial or financial relationships that might be construed as a potential conflict of interest.

## Footnotes

1 https://github.com/PacificBiosciences/barcoding

2 http://cutadapt.readthedocs.io/en/stable/

3 http://www.drive5.com/usearch/manual/uchime_algo.html

4 http://sourceforge.net/projects/rdpclassifier/

5 https://www.arb-silva.de/

6 http://biocore.github.io/pynast/

7 http://ab.inf.uni-tuebingen.de/software/megan5/
