# Supplementary material for: Fermented Soybean Meal Replacement in the Diet of Lactating Holstein Dairy Cows: Modulated Rumen Fermentation and Ruminal Microflora
Source: Front Microbiol. 2021 Jan 29;12:625857. doi: 10.3389/fmicb.2021.625857 (PMC7879537; doi:10.3389/fmicb.2021.625857)
Supplement: Supplementary file 2 [file Image_2.PDF]

Supplementary Material

Fermented soybean meal replacement in the diet of lactating Holstein dairy cows: improved rumen fermentation and modulated ruminal microflora

Zuo Wang [1], Yuannian Yu [1], Xinyao Li [1], Hongyan Xiao [1], Peihua Zhang [1], Weijun Shen [1], Fachun Wan [1], Jianhua He [1], Hui Yao [3], Duanqin Wu [4]*, Zhiliang Tan [2], and Shaoxun Tang [2]*

[1] College of Animal Science and Technology, Hunan Agricultural University, Changsha, Hunan 410128, China

[2] CAS Key Laboratory of Agro-Ecological Processes in Subtropical Region, National Engineering Laboratory for Pollution Control and Waste Utilization in Livestock and Poultry Production, Hunan Provincial Key Laboratory of Animal Nutrition & Physiology and Metabolism, Institute of Subtropical Agriculture, Chinese Academy of Sciences, Changsha, Hunan 410125, China

[3] Nanshan Dairy Co. Ltd., Shaoyang, Hunan 422500, China

[4] Institute of Bast Fiber Crops, Chinese Academy of Agricultural Sciences, Changsha, Hunan, 410205, China

*** Correspondence:**

Shaoxun Tang; Duanqin Wu

shaoxuntang@163.com; wuduanqin@caas.cn

Supplementary Figures

Figure S2. Relative abundances of the top 10 bacterial taxa at the phylum level across samples

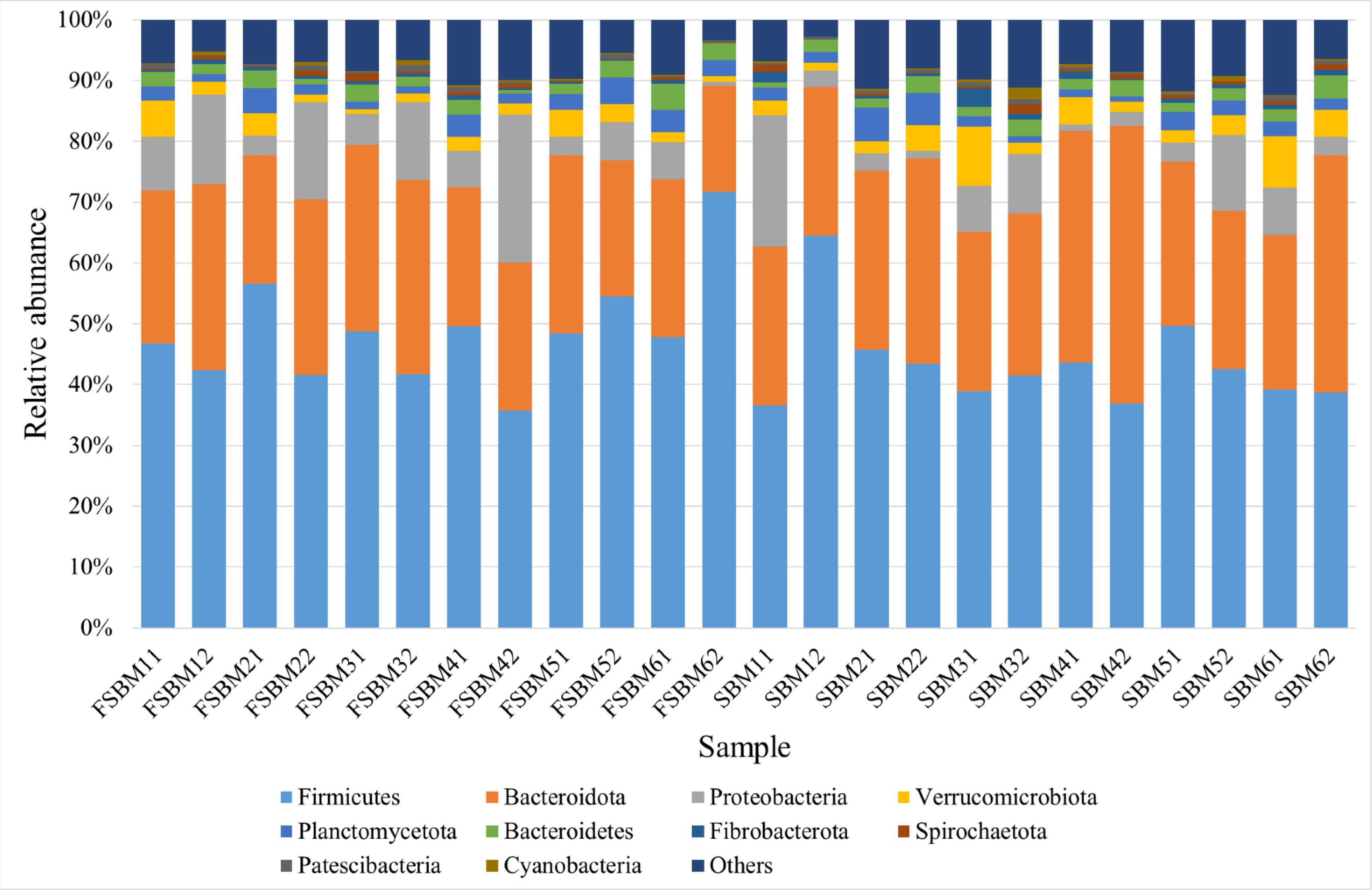

100%
90%
80%
70%
60%
50%
40%
30%
20%
10%
0%
Relative abunance
FSBM11
FSBM12
FSBM21
FSBM22
FSBM31
FSBM32
FSBM41
FSBM42
FSBM51
FSBM52
FSBM61
FSBM62
SBM11
SBM12
SBM21
SBM22
SBM31
SBM32
SBM41
SBM42
SBM51
SBM52
SBM61
SBM62
Sample
Firmicutes
Bacteroidota
Proteobacteria
Verrucomicrobiota
Planctomycetota
Bacteroidetes
Fibrobacterota
Spirochaetota
Patescibacteria
Cyanobacteria
Others
