# Supplementary material for: Fermented Soybean Meal Replacement in the Diet of Lactating Holstein Dairy Cows: Modulated Rumen Fermentation and Ruminal Microflora
Source: Front Microbiol. 2021 Jan 29;12:625857. doi: 10.3389/fmicb.2021.625857 (PMC7879537; doi:10.3389/fmicb.2021.625857)
Supplement: Supplementary file 4 [file Image_4.PDF]

Supplementary Material

Fermented soybean meal replacement in the diet of lactating Holstein dairy cows: improved rumen fermentation and modulated ruminal microflora

Zuo Wang [1], Yuannian Yu [1], Xinyao Li [1], Hongyan Xiao [1], Peihua Zhang [1], Weijun Shen [1], Fachun Wan [1], Jianhua He [1], Hui Yao [3], Duanqin Wu [4]*, Zhiliang Tan [2], and Shaoxun Tang [2]*

[1] College of Animal Science and Technology, Hunan Agricultural University, Changsha, Hunan 410128, China

[2] CAS Key Laboratory of Agro-Ecological Processes in Subtropical Region, National Engineering Laboratory for Pollution Control and Waste Utilization in Livestock and Poultry Production, Hunan Provincial Key Laboratory of Animal Nutrition & Physiology and Metabolism, Institute of Subtropical Agriculture, Chinese Academy of Sciences, Changsha, Hunan 410125, China

[3] Nanshan Dairy Co. Ltd., Shaoyang, Hunan 422500, China

[4] Institute of Bast Fiber Crops, Chinese Academy of Agricultural Sciences, Changsha, Hunan, 410205, China

*** Correspondence:**

Shaoxun Tang; Duanqin Wu

shaoxuntang@163.com; wuduanqin@caas.cn

Supplementary Figures

Figure S4. Relative abundances of the top 10 bacterial taxa at the species level across samples

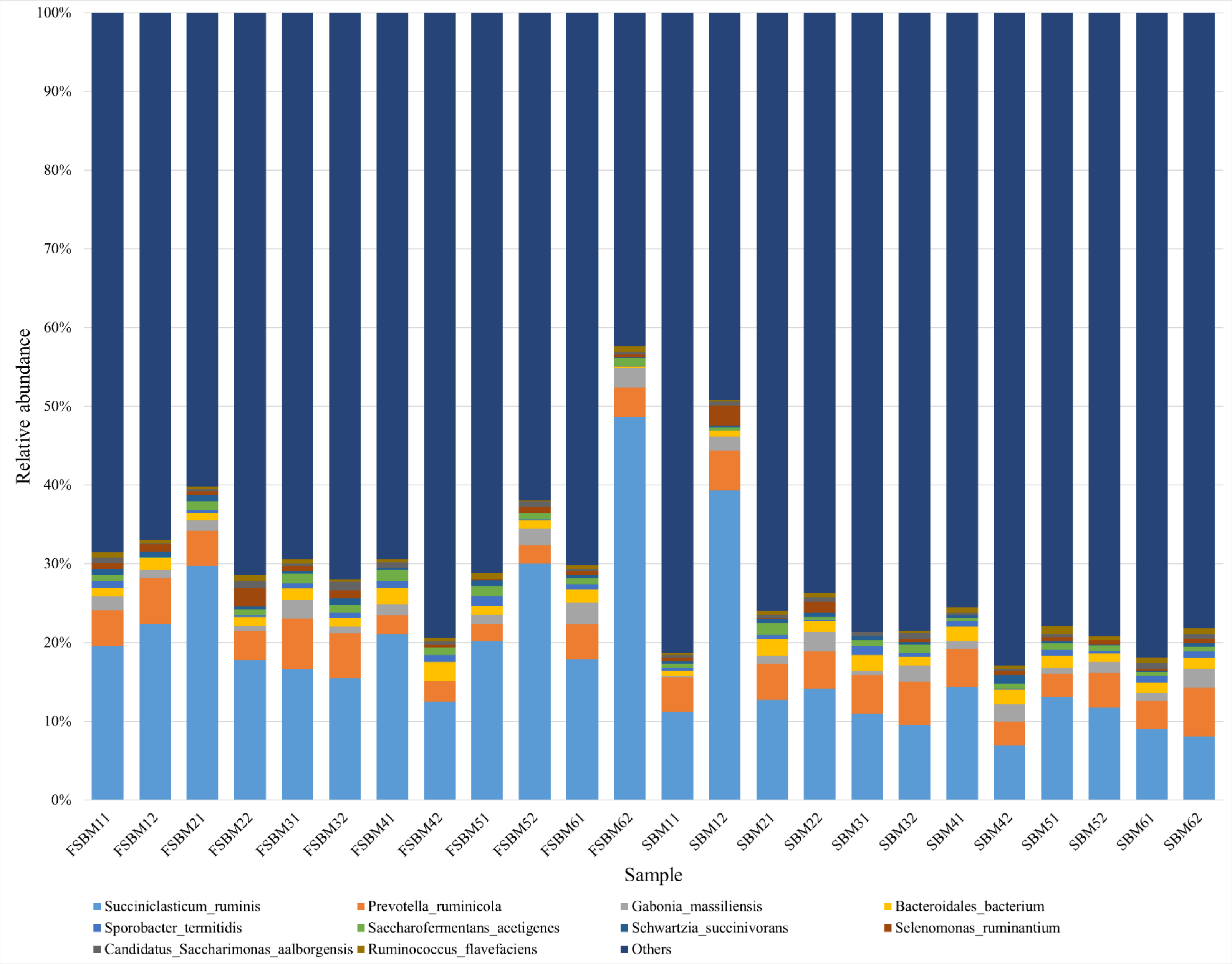
100%
90%
80%
70%
60%
50%
40%
30%
20%
10%
0%
Relative abundance
FSBM11
FSBM12
FSBM21
FSBM22
FSBM31
FSBM32
FSBM41
FSBM42
FSBM51
FSBM52
FSBM61
FSBM62
SBM11
SBM12
SBM21
SBM22
SBM31
SBM32
SBM41
SBM42
SBM51
SBM52
SBM61
SBM62
Sample
Succiniclasticum_ruminis
Prevotella_ruminicola
Gabonia_massiliensis
Bacteroidales_bacterium
Sporobacter_termitidis
Saccharofermentans_acetigenes
Schwartzia_succinivorans
Selenomonas_ruminantium
Candidatus_Saccharimonas_aalborgensis
Ruminococcus_flavefaciens
Others
