# Supplementary material for: Fermented Soybean Meal Replacement in the Diet of Lactating Holstein Dairy Cows: Modulated Rumen Fermentation and Ruminal Microflora
Source: Front Microbiol. 2021 Jan 29;12:625857. doi: 10.3389/fmicb.2021.625857 (PMC7879537; doi:10.3389/fmicb.2021.625857)
Supplement: Supplementary file 5 [file Table_1.pdf]

Supplementary Material

Fermented soybean meal replacement in the diet of lactating Holstein dairy cows: improved rumen fermentation and modulated ruminal microflora

Zuo Wang [1], Yuannian Yu [1], Xinyao Li [1], Hongyan Xiao [1], Peihua Zhang [1], Weijun Shen [1], Fachun Wan [1], Jianhua He [1], Shaoxun Tang [2]*, Zhiliang Tan [2], Duanqin Wu [3]*, and Hui Yao [4]

[1] College of Animal Science and Technology, Hunan Agricultural University, Changsha, Hunan 410128, China

[2] CAS Key Laboratory of Agro-Ecological Processes in Subtropical Region, National Engineering Laboratory for Pollution Control and Waste Utilization in Livestock and Poultry Production, Hunan Provincial Key Laboratory of Animal Nutrition & Physiology and Metabolism, Institute of Subtropical Agriculture, Chinese Academy of Sciences, Changsha, Hunan 410125, China

[3] Institute of Bast Fiber Crops, Chinese Academy of Agricultural Sciences, Changsha, Hunan, 410205, China

[4] Nanshan Dairy Co. Ltd., Shaoyang, Hunan 422500, China

*** Correspondence:**

Shaoxun Tang; Duanqin Wu

shaoxuntang@163.com; wuduanqin@caas.cn

Supplementary Tables

Table S1. Summary of sequencing data

Sample ID	Barcode-CCS	Filtered-CCS	Optimization-CCS	AvgLen (bp)	Effective (%)	OTU	ACE	Chao1	Simpson	Shannon	Coverage
SBM11	5194	5183	5062	1454	97.46	265	387.7644	392.8333	0.048086	4.439725	0.887939
SBM12	7516	7507	7245	1459	96.39	188	322.0045	330.6	0.130684	3.567814	0.911681
SBM21	4817	4813	4715	1453	97.88	308	418.6684	394.6234	0.016323	5.100524	0.889839
SBM22	6343	6335	6182	1455	97.46	286	444.2441	433.7818	0.019705	4.893841	0.878443
SBM31	5077	5066	4965	1453	97.79	285	410.398	375.4	0.013971	4.991234	0.892688
SBM32	5200	5193	5109	1454	98.25	330	484.5157	479.1429	0.015263	5.135577	0.862298
SBM41	4689	4678	4593	1454	97.95	270	385.4848	366.6935	0.027443	4.700536	0.895537
SBM42	5338	5333	5194	1454	97.3	269	393.4226	407.4898	0.026782	4.703255	0.888889
SBM51	4990	4979	4858	1453	97.35	324	459.5925	434.3289	0.013192	5.183006	0.876543
SBM52	4648	4636	4514	1455	97.12	306	466.8098	451.8871	0.021057	4.938149	0.871795
SBM61	4470	4457	4391	1454	98.23	289	437.9036	427.1579	0.012951	5.013911	0.880342
SBM62	4948	4934	4857	1453	98.16	295	421.7785	406.5625	0.014319	5.020057	0.88604
FSBM11	6081	6069	5908	1456	97.16	276	423.6805	408.2222	0.035259	4.669754	0.88604
FSBM12	7306	7296	7139	1455	97.71	258	557.0745	434.449	0.059479	4.234471	0.874644
FSBM21	5057	5048	4872	1458	96.34	250	345.1942	341.5283	0.062826	4.377213	0.905983
FSBM22	4918	4912	4717	1457	95.91	259	380.1349	374.0545	0.042293	4.473073	0.892688
FSBM31	5284	5281	5130	1454	97.09	285	419.624	412.5	0.024202	4.887298	0.88604
FSBM32	5370	5364	5221	1456	97.23	288	436.176	397.1549	0.027531	4.754302	0.881292
FSBM41	5328	5325	5196	1454	97.52	311	471.8374	440.3889	0.032671	4.835633	0.869896
FSBM42	4479	4472	4379	1453	97.77	277	399.3025	373.1781	0.063857	4.373642	0.88699
FSBM51	4821	4818	4729	1455	98.09	295	476.6768	457.5593	0.029586	4.767638	0.867996
FSBM52	4171	4159	4015	1458	96.26	251	383.7051	360.0526	0.055243	4.303387	0.893637
FSBM61	4827	4822	4712	1454	97.62	275	381.5232	360.3125	0.029331	4.792156	0.900285
FSBM62	4792	4788	4614	1462	96.29	211	369.6585	347.8837	0.178075	3.410464	0.896486
