# Supplementary material for: Fermented Soybean Meal Replacement in the Diet of Lactating Holstein Dairy Cows: Modulated Rumen Fermentation and Ruminal Microflora
Source: Front Microbiol. 2021 Jan 29;12:625857. doi: 10.3389/fmicb.2021.625857 (PMC7879537; doi:10.3389/fmicb.2021.625857)
Supplement: Supplementary file 6 [file Table_2.pdf]

Supplementary Material

Fermented soybean meal replacement in the diet of lactating Holstein dairy cows: improved rumen fermentation and modulated ruminal microflora

Zuo Wang [1], Yuannian Yu [1], Xinyao Li [1], Hongyan Xiao [1], Peihua Zhang [1], Weijun Shen [1], Fachun Wan [1], Jianhua He [1], Shaoxun Tang [2]*, Zhiliang Tan [2], Duanqin Wu [3]*, and Hui Yao [4]

[1] College of Animal Science and Technology, Hunan Agricultural University, Changsha, Hunan 410128, China

[2] CAS Key Laboratory of Agro-Ecological Processes in Subtropical Region, National Engineering Laboratory for Pollution Control and Waste Utilization in Livestock and Poultry Production, Hunan Provincial Key Laboratory of Animal Nutrition & Physiology and Metabolism, Institute of Subtropical Agriculture, Chinese Academy of Sciences, Changsha, Hunan 410125, China

[3] Institute of Bast Fiber Crops, Chinese Academy of Agricultural Sciences, Changsha, Hunan, 410205, China

[4] Nanshan Dairy Co. Ltd., Shaoyang, Hunan 422500, China

*** Correspondence:**

Shaoxun Tang; Duanqin Wu

shaoxuntang@163.com; wuduanqin@caas.cn

Supplementary Tables

Table S2. OTU abundances and taxonomic annotation across treatments

OTU_ID	SBM	FSBM	Taxonomy
OTU1	1313	2254	k__Bacteria; p__Firmicutes; c__Negativicutes; o__Acidaminococcales; f__Acidaminococcaceae; g__Succiniclasticum; s__Succiniclasticum_ruminis
OTU10	35	45	k__Bacteria; p__Proteobacteria; c__Gammaproteobacteria; o__Aeromonadales; f__Succinivibrionaceae; g__Succinivibrio; s__Succinivibrio_dextrinosolvens

OTU100	84	72	k__Bacteria; p__Firmicutes; c__Negativicutes; o__Veillonellales-Selenomonadales; f__Selenomonadaceae; Unclassified; Unclassified
OTU101	55	40	k__Bacteria; Unclassified; Unclassified; Unclassified; Unclassified; Unclassified; Unclassified
OTU102	6	9	k__Bacteria; p__Bacteroidota; c__Bacteroidia; o__Bacteroidales; f__Prevotellaceae; g__Prevotella; Unclassified
OTU104	95	106	k__Bacteria; p__Bacteroidota; c__Bacteroidia; o__Bacteroidales; f__Prevotellaceae; g__Prevotella; s__Prevotella_ruminicola
OTU105	42	28	k__Bacteria; Unclassified; Unclassified; Unclassified; Unclassified; Unclassified; Unclassified
OTU106	18	10	k__Bacteria; p__Firmicutes; c__Clostridia; o__Clostridiales; f__Hungateiclostridiaceae; g__Saccharofermentans; s__Saccharofermentans_acetigenes
OTU107	51	54	k__Bacteria; p__Firmicutes; c__Negativicutes; o__Veillonellales-Selenomonadales; f__Selenomonadaceae; g__Schwartzia; s__Schwartzia_succinivorans
OTU109	113	102	k__Bacteria; p__Bacteroidota; c__Bacteroidia; o__Bacteroidales; f__Prevotellaceae; g__Prevotella; s__Prevotella_ruminicola
OTU11	25	20	k__Bacteria; p__Planctomycetota; c__Planctomycetes; o__Pirellulales; f__Pirellulaceae; Unclassified; Unclassified
OTU110	37	37	k__Bacteria; p__Firmicutes; c__Clostridia; Unclassified; Unclassified; Unclassified; Unclassified
OTU111	6	13	k__Bacteria; p__Bacteroidota; c__Bacteroidia; o__Bacteroidales; f__Prevotellaceae; g__Prevotella; s__Prevotella_sp
OTU112	39	29	k__Bacteria; p__Firmicutes; c__Bacilli; o__Acholeplasmatales; f__Acholeplasmataceae; g__Anaeroplasma; s__Anaeroplasma_varium
OTU113	110	165	k__Bacteria; p__Bacteroidota; c__Bacteroidia; o__Bacteroidales; f__Prevotellaceae; g__Prevotella; Unclassified
OTU114	36	44	k__Bacteria; p__Firmicutes; c__Clostridia; o__Oscillospirales; f__Oscillospiraceae; g__Sporobacter; s__Sporobacter_termitidis
OTU115	31	37	k__Bacteria; p__Firmicutes; c__Clostridia; o__Clostridiales; f__Hungateiclostridiaceae; g__Saccharofermentans; s__Saccharofermentans_acetigenes
OTU116	33	36	k__Bacteria; p__Bacteroidota; c__Bacteroidia; Unclassified; Unclassified; Unclassified; Unclassified
OTU117	3	5	k__Bacteria; p__Firmicutes; c__Clostridia; Unclassified; Unclassified; Unclassified; Unclassified
OTU118	22	35	k__Bacteria; p__Firmicutes; c__Clostridia; o__Clostridiales; f__Hungateiclostridiaceae; g__Saccharofermentans; s__Saccharofermentans_acetigenes
OTU119	28	48	k__Bacteria; p__Desulfobacterota; c__Desulfovibrionia; o__Desulfovibrionales; f__Desulfovibrionaceae; g__Desulfovibrio; Unclassified
OTU12	123	5	k__Bacteria; p__Bacteroidota; c__Bacteroidia; o__Bacteroidales;

			f__Prevotellaceae; g__Prevotella; s__Prevotella_sp
OTU120	5	5	k__Bacteria; Unclassified; Unclassified; Unclassified; Unclassified; Unclassified; Unclassified
OTU121	13	4	k__Bacteria; p__Firmicutes; Unclassified; Unclassified; Unclassified; Unclassified; Unclassified
OTU122	7	3	k__Bacteria; p__Firmicutes; c__Clostridia; o__Oscillospirales; f__Oscillospiraceae; Unclassified; Unclassified
OTU123	81	79	k__Bacteria; p__Bacteroidota; c__Bacteroidia; o__Bacteroidales; f__Prevotellaceae; g__Prevotella; s__Prevotella_ruminicola
OTU124	7	9	k__Bacteria; p__Firmicutes; c__Clostridia; o__Lachnospirales; f__Lachnospiraceae; Unclassified; Unclassified
OTU125	12	7	k__Bacteria; p__Firmicutes; c__Clostridia; o__Eubacteriales; f__Eubacteriaceae; g__Eubacterium; s__Eubacterium_ruminantium
OTU126	48	29	k__Bacteria; p__Bacteroidota; c__Bacteroidia; o__Bacteroidales; Unclassified; Unclassified; Unclassified
OTU127	4	15	k__Bacteria; p__Firmicutes; c__Clostridia; o__Clostridiales; f__Hungateiclostridiaceae; g__Saccharofermentans; s__Saccharofermentans_acetigenes
OTU128	6	13	k__Bacteria; p__Planctomycetota; c__Planctomycetes; o__Pirellulales; f__Pirellulaceae; Unclassified; Unclassified
OTU129	50	44	k__Bacteria; p__Bacteroidota; c__Bacteroidia; o__Bacteroidales; f__Prevotellaceae; g__Prevotella; s__Prevotella_ruminicola
OTU13	29	17	k__Bacteria; p__Firmicutes; Unclassified; Unclassified; Unclassified; Unclassified; Unclassified
OTU130	5	5	k__Bacteria; p__Firmicutes; c__Clostridia; Unclassified; Unclassified; Unclassified; Unclassified
OTU131	26	17	k__Bacteria; Unclassified; Unclassified; Unclassified; Unclassified; Unclassified; Unclassified
OTU132	2	6	k__Bacteria; p__Firmicutes; c__Clostridia; Unclassified; Unclassified; Unclassified; Unclassified
OTU133	34	68	k__Bacteria; p__Bacteroidota; c__Bacteroidia; o__Bacteroidales; f__Prevotellaceae; g__Prevotella; Unclassified
OTU134	27	24	k__Bacteria; p__Firmicutes; c__Bacilli; Unclassified; Unclassified; Unclassified; Unclassified
OTU136	11	6	k__Bacteria; p__Cyanobacteria; Unclassified; Unclassified; Unclassified; Unclassified; Unclassified
OTU137	14	17	k__Bacteria; Unclassified; Unclassified; Unclassified; Unclassified; Unclassified; Unclassified
OTU138	11	12	k__Bacteria; p__Firmicutes; c__Clostridia; Unclassified; Unclassified; Unclassified; Unclassified
OTU139	14	15	k__Bacteria; p__Firmicutes; c__Clostridia; o__Clostridiales; Unclassified; Unclassified; Unclassified
OTU14	50	46	k__Bacteria; p__Proteobacteria; c__Gammaproteobacteria; o__Aeromonadales; f__Succinivibrionaceae; g__Succinivibrio;

			s__Succinivibrio_dextrinosolvens
OTU140	4	15	k__Bacteria; p__Firmicutes; c__Clostridia; Unclassified; Unclassified; Unclassified; Unclassified
OTU141	6	2	k__Bacteria; p__Firmicutes; c__Bacilli; o__Acholeplasmatales; f__Acholeplasmataceae; g__Anaeroplasma; Unclassified
OTU142	28	13	k__Bacteria; p__Bacteroidota; c__Bacteroidia; o__Bacteroidales; f__Prevotellaceae; g__Prevotella; Unclassified
OTU143	6	6	k__Bacteria; Unclassified; Unclassified; Unclassified; Unclassified; Unclassified; Unclassified
OTU144	16	26	k__Bacteria; p__Firmicutes; c__Clostridia; o__Eubacteriales; f__Eubacteriaceae; g__Eubacterium; s__Eubacterium_ruminantium
OTU145	25	24	k__Bacteria; p__Firmicutes; Unclassified; Unclassified; Unclassified; Unclassified; Unclassified
OTU146	17	13	k__Bacteria; p__Firmicutes; c__Clostridia; o__Eubacteriales; f__Eubacteriaceae; g__Eubacterium; s__Eubacterium_ruminantium
OTU147	13	8	k__Bacteria; p__Firmicutes; c__Bacilli; o__Acholeplasmatales; f__Acholeplasmataceae; Unclassified; Unclassified
OTU148	17	5	k__Bacteria; p__Firmicutes; Unclassified; Unclassified; Unclassified; Unclassified; Unclassified
OTU149	11	8	k__Bacteria; p__Firmicutes; c__Clostridia; o__Eubacteriales; f__Eubacteriaceae; g__Eubacterium; s__Eubacterium_ruminantium
OTU15	178	75	k__Bacteria; p__Bacteroidota; c__Bacteroidia; o__Bacteroidales; f__Prevotellaceae; g__Prevotella; s__Prevotella_sp
OTU150	6	4	k__Bacteria; p__Proteobacteria; c__Alphaproteobacteria; Unclassified; Unclassified; Unclassified; Unclassified
OTU151	11	8	k__Bacteria; p__Bacteroidota; c__Bacteroidia; o__Bacteroidales; f__Prevotellaceae; g__Prevotella; s__Prevotella_sp
OTU152	6	2	k__Bacteria; p__Cyanobacteria; Unclassified; Unclassified; Unclassified; Unclassified; Unclassified
OTU153	7	6	k__Bacteria; p__Firmicutes; c__Clostridia; o__Oscillospirales; f__Oscillospiraceae; Unclassified; Unclassified
OTU154	6	4	k__Bacteria; p__Firmicutes; c__Bacilli; o__Acholeplasmatales; f__Acholeplasmataceae; g__Anaeroplasma; Unclassified
OTU155	3	2	k__Bacteria; p__Firmicutes; c__Clostridia; o__Clostridiales; f__Hungateiclostridiaceae; g__Saccharofermentans; s__Saccharofermentans_acetigenes
OTU156	12	13	k__Bacteria; Unclassified; Unclassified; Unclassified; Unclassified; Unclassified; Unclassified
OTU157	101	47	k__Bacteria; p__Fibrobacterota; c__Fibrobacteria; o__Fibrobacterales; f__Fibrobacteraceae; g__Fibrobacter; s__Fibrobacter_succinogenes
OTU158	4	8	k__Bacteria; p__Firmicutes; c__Clostridia; o__Clostridiales; f__Caloramatoraceae; Unclassified; Unclassified
OTU159	103	88	k__Bacteria; Unclassified; Unclassified; Unclassified; Unclassified; Unclassified; Unclassified

OTU16	209	169	k__Bacteria; p__Bacteroidota; c__Bacteroidia; o__Bacteroidales; f__Prevotellaceae; g__Prevotella; s__Prevotella_sp
OTU160	3	2	k__Bacteria; p__Bacteroidota; c__Bacteroidia; o__Bacteroidales; f__Prevotellaceae; g__Prevotella; Unclassified
OTU161	15	17	k__Bacteria; p__Firmicutes; c__Clostridia; o__Oscillospirales; f__Oscillospiraceae; Unclassified; Unclassified
OTU162	33	30	k__Bacteria; p__Bacteroidota; c__Bacteroidia; o__Bacteroidales; f__Prevotellaceae; g__Prevotella; s__Prevotella_sp
OTU163	5	6	k__Bacteria; p__Firmicutes; c__Clostridia; o__Lachnospirales; f__Lachnospiraceae; Unclassified; Unclassified
OTU164	33	51	k__Bacteria; Unclassified; Unclassified; Unclassified; Unclassified; Unclassified; Unclassified
OTU165	3	5	k__Bacteria; p__Firmicutes; c__Clostridia; Unclassified; Unclassified; Unclassified; Unclassified
OTU167	5	1	k__Bacteria; p__Verrucomicrobiota; c__Verrucomicrobiae; Unclassified; Unclassified; Unclassified; Unclassified
OTU168	7	18	k__Bacteria; p__Firmicutes; c__Clostridia; Unclassified; Unclassified; Unclassified; Unclassified
OTU169	6	11	k__Bacteria; Unclassified; Unclassified; Unclassified; Unclassified; Unclassified; Unclassified
OTU17	13	16	k__Bacteria; p__Firmicutes; c__Clostridia; Unclassified; Unclassified; Unclassified; Unclassified
OTU170	12	15	k__Bacteria; p__Firmicutes; c__Bacilli; Unclassified; Unclassified; Unclassified; Unclassified
OTU171	10	8	k__Bacteria; p__Bacteroidota; c__Bacteroidia; o__Bacteroidales; f__Prevotellaceae; g__Prevotella; Unclassified
OTU172	17	31	k__Bacteria; p__Firmicutes; c__Clostridia; Unclassified; Unclassified; Unclassified; Unclassified
OTU173	7	5	k__Bacteria; p__Firmicutes; c__Bacilli; Unclassified; Unclassified; Unclassified; Unclassified
OTU174	15	15	k__Bacteria; p__Firmicutes; c__Bacilli; o__Acholeplasmatales; f__Acholeplasmataceae; g__Anaeroplasma; Unclassified
OTU175	2	3	k__Bacteria; p__Firmicutes; c__Clostridia; o__Oscillospirales; f__Ruminococcaceae; Unclassified; Unclassified
OTU176	12	8	k__Bacteria; p__Cyanobacteria; Unclassified; Unclassified; Unclassified; Unclassified; Unclassified
OTU177	6	13	k__Bacteria; p__Patescibacteria; c__Saccharimonadia; o__Saccharimonadales; f__Saccharimonadaceae; g__Candidatus_Saccharimonas; s__Candidatus_Saccharimonas_aalborgensis
OTU178	5	6	k__Bacteria; p__Firmicutes; c__Clostridia; o__Christensenellales; f__Christensenellaceae; g__Christensenella; s__Christensenella_sp
OTU179	25	16	k__Bacteria; p__Firmicutes; c__Clostridia; o__Oscillospirales; f__Oscillospiraceae; Unclassified; Unclassified

OTU18	18	17	k__Bacteria; p__Bacteroidota; c__Bacteroidia; o__Bacteroidales; Unclassified; Unclassified; Unclassified
OTU180	4	4	k__Bacteria; p__Firmicutes; c__Bacilli; Unclassified; Unclassified; Unclassified; Unclassified
OTU181	2	5	k__Bacteria; p__Proteobacteria; c__Gammaproteobacteria; o__Xanthomonadales; f__Xanthomonadaceae; g__Stenotrophomonas; s__Stenotrophomonas_rhizophila
OTU182	6	10	k__Bacteria; p__Firmicutes; c__Clostridia; o__Clostridiales; f__Caloramatoraceae; Unclassified; Unclassified
OTU183	4	12	k__Bacteria; p__Firmicutes; c__Clostridia; o__Lachnospirales; f__Lachnospiraceae; Unclassified; Unclassified
OTU184	2	7	k__Bacteria; p__Firmicutes; c__Bacilli; Unclassified; Unclassified; Unclassified; Unclassified
OTU185	35	31	k__Bacteria; Unclassified; Unclassified; Unclassified; Unclassified; Unclassified; Unclassified
OTU186	22	35	k__Bacteria; p__Bacteroidetes; c__Bacteroidia; o__Bacteroidales; f__Prevotellaceae; g__Marseilla; s__Marseilla_massiliensis
OTU187	22	32	k__Bacteria; p__Bacteroidota; c__Bacteroidia; o__Bacteroidales; f__Prevotellaceae; g__Prevotella; Unclassified
OTU188	42	52	k__Bacteria; p__Firmicutes; c__Clostridia; o__Oscillospirales; f__Oscillospiraceae; Unclassified; Unclassified
OTU189	6	11	k__Bacteria; p__Firmicutes; c__Clostridia; o__Clostridiales; f__Hungateiclostridiaceae; g__Saccharofermentans; s__Saccharofermentans_acetigenes
OTU19	22	23	k__Bacteria; p__Firmicutes; c__Clostridia; Unclassified; Unclassified; Unclassified; Unclassified
OTU190	59	57	k__Bacteria; p__Bacteroidota; c__Bacteroidia; o__Bacteroidales; f__Prevotellaceae; g__Prevotella; s__Prevotella_bryantii
OTU191	8	5	k__Bacteria; p__Firmicutes; c__Clostridia; Unclassified; Unclassified; Unclassified; Unclassified
OTU192	20	19	k__Bacteria; Unclassified; Unclassified; Unclassified; Unclassified; Unclassified; Unclassified
OTU193	26	31	k__Bacteria; p__Firmicutes; c__Clostridia; o__Lachnospirales; f__Lachnospiraceae; Unclassified; Unclassified
OTU194	28	7	k__Bacteria; p__Verrucomicrobiota; c__Verrucomicrobiae; Unclassified; Unclassified; Unclassified; Unclassified
OTU195	35	38	k__Bacteria; p__Firmicutes; c__Clostridia; o__Oscillospirales; f__Ruminococcaceae; g__Ruminococcus; s__Ruminococcus_flavefaciens
OTU196	11	14	k__Bacteria; p__Firmicutes; c__Clostridia; Unclassified; Unclassified; Unclassified; Unclassified
OTU197	13	13	k__Bacteria; p__Firmicutes; c__Bacilli; o__Lactobacillales; f__Lactobacillaceae; g__Lactobacillus; s__Lactobacillus_fermentum
OTU198	16	17	k__Bacteria; p__Firmicutes; c__Clostridia; o__Clostridiales; Unclassified; Unclassified; Unclassified

OTU199	90	78	k__Bacteria; p__Bacteroidota; c__Bacteroidia; o__Bacteroidales; f__Prevotellaceae; g__Prevotella; s__Prevotella_ruminicola
OTU2	58	48	k__Bacteria; p__Proteobacteria; c__Alphaproteobacteria; o__Acetobacterales; f__Acetobacteraceae; g__Acetobacter; s__Acetobacter_pasteurianus
OTU20	42	6	k__Bacteria; p__Verrucomicrobiota; Unclassified; Unclassified; Unclassified; Unclassified; Unclassified
OTU200	3	4	k__Bacteria; p__Firmicutes; c__Bacilli; o__Lactobacillales; f__Lactobacillaceae; g__Lactobacillus; s__Lactobacillus_amylovorus
OTU201	11	13	k__Bacteria; p__Firmicutes; c__Clostridia; o__Oscillospirales; f__Ruminococcaceae; g__Ruminococcus; s__Ruminococcus_flavefaciens
OTU202	57	73	k__Bacteria; p__Bacteroidetes; c__Bacteroidia; o__Bacteroidales; f__Porphyromonadaceae; g__Gabonia; s__Gabonia_massiliensis
OTU205	3	1	k__Bacteria; p__Campilobacterota; c__Campylobacteria; o__Campylobacterales; f__Campylobacteraceae; g__Campylobacter; s__Campylobacter_fetus
OTU206	10	8	k__Bacteria; p__Firmicutes; c__Clostridia; Unclassified; Unclassified; Unclassified; Unclassified
OTU207	13	14	k__Bacteria; p__Firmicutes; c__Bacilli; Unclassified; Unclassified; Unclassified; Unclassified
OTU208	9	8	k__Bacteria; p__Verrucomicrobiota; Unclassified; Unclassified; Unclassified; Unclassified; Unclassified
OTU209	18	14	k__Bacteria; p__Patescibacteria; c__Saccharimonadia; o__Saccharimonadales; f__Saccharimonadaceae; g__Candidatus_Saccharimonas; s__Candidatus_Saccharimonas_aalborgensis
OTU21	39	30	k__Bacteria; p__Firmicutes; Unclassified; Unclassified; Unclassified; Unclassified; Unclassified
OTU210	5	2	k__Bacteria; Unclassified; Unclassified; Unclassified; Unclassified; Unclassified; Unclassified
OTU212	7	4	k__Bacteria; p__Firmicutes; c__Bacilli; Unclassified; Unclassified; Unclassified; Unclassified
OTU213	4	9	k__Bacteria; p__Bacteroidota; c__Bacteroidia; o__Bacteroidales; f__Prevotellaceae; g__Prevotella; s__Prevotella_sp
OTU214	13	11	k__Bacteria; p__Firmicutes; c__Clostridia; Unclassified; Unclassified; Unclassified; Unclassified
OTU215	4	3	k__Bacteria; p__Firmicutes; c__Clostridia; Unclassified; Unclassified; Unclassified; Unclassified
OTU216	9	8	k__Bacteria; p__Firmicutes; Unclassified; Unclassified; Unclassified; Unclassified; Unclassified
OTU218	10	11	k__Bacteria; p__Bacteroidota; c__Bacteroidia; o__Bacteroidales; f__Rikenellaceae; g__Bact; s__Bacteroidales_bacterium
OTU219	0	6	k__Bacteria; Unclassified; Unclassified; Unclassified; Unclassified; Unclassified; Unclassified

OTU22	54	57	k__Bacteria; p__Planctomycetota; c__Planctomycetes; o__Pirellulales; f__Pirellulaceae; Unclassified; Unclassified
OTU220	6	4	k__Bacteria; p__Verrucomicrobiota; Unclassified; Unclassified; Unclassified; Unclassified; Unclassified
OTU221	8	12	k__Bacteria; p__Firmicutes; c__Clostridia; o__Clostridiales; f__Hungateiclostridiaceae; Unclassified; Unclassified
OTU222	10	14	k__Bacteria; Unclassified; Unclassified; Unclassified; Unclassified; Unclassified; Unclassified
OTU223	20	7	k__Bacteria; Unclassified; Unclassified; Unclassified; Unclassified; Unclassified; Unclassified
OTU224	13	20	k__Bacteria; p__Patescibacteria; c__Saccharimonadia; o__Saccharimonadales; f__Saccharimonadaceae; g__Candidatus_Saccharimonas; s__Candidatus_Saccharimonas_aalborgensis
OTU225	74	83	k__Bacteria; p__Bacteroidetes; c__Bacteroidia; o__Bacteroidales; f__Porphyromonadaceae; g__Gabonia; s__Gabonia_massiliensis
OTU226	5	1	k__Bacteria; p__Firmicutes; c__Clostridia; o__Eubacteriales; f__Eubacteriaceae; g__Eubacterium; s__Eubacterium_ruminantium
OTU227	5	3	k__Bacteria; p__Firmicutes; c__Bacilli; Unclassified; Unclassified; Unclassified; Unclassified
OTU228	23	6	k__Bacteria; p__Bacteroidota; c__Bacteroidia; o__Bacteroidales; f__Prevotellaceae; g__Prevotella; Unclassified
OTU229	4	2	k__Bacteria; p__Proteobacteria; c__Alphaproteobacteria; Unclassified; Unclassified; Unclassified; Unclassified
OTU23	15	12	k__Bacteria; p__Proteobacteria; c__Alphaproteobacteria; Unclassified; Unclassified; Unclassified; Unclassified
OTU230	9	22	k__Bacteria; p__Firmicutes; c__Clostridia; o__Eubacteriales; f__Eubacteriaceae; Unclassified; Unclassified
OTU231	10	8	k__Bacteria; Unclassified; Unclassified; Unclassified; Unclassified; Unclassified; Unclassified
OTU232	12	12	k__Bacteria; p__Firmicutes; c__Clostridia; o__Lachnospirales; f__Lachnospiraceae; Unclassified; Unclassified
OTU233	10	10	k__Bacteria; p__Firmicutes; c__Bacilli; Unclassified; Unclassified; Unclassified; Unclassified
OTU235	6	6	k__Bacteria; p__Firmicutes; c__Bacilli; Unclassified; Unclassified; Unclassified; Unclassified
OTU236	13	10	k__Bacteria; p__Firmicutes; c__Clostridia; o__Lachnospirales; f__Lachnospiraceae; g__Butyrivibrio; s__Butyrivibrio_fibrisolvens
OTU237	23	13	k__Bacteria; p__Bacteroidota; c__Bacteroidia; o__Bacteroidales; f__Prevotellaceae; g__Prevotella; Unclassified
OTU238	9	9	k__Bacteria; p__Firmicutes; c__Bacilli; Unclassified; Unclassified; Unclassified; Unclassified
OTU239	14	5	k__Bacteria; p__Firmicutes; c__Negativicutes; o__Veillonellales-Selenomonadales; f__Selenomonadaceae; Unclassified; Unclassified

OTU24	30	6	k__Bacteria; p__Firmicutes; c__Bacilli; Unclassified; Unclassified; Unclassified; Unclassified
OTU240	6	0	k__Bacteria; p__Firmicutes; Unclassified; Unclassified; Unclassified; Unclassified; Unclassified
OTU241	12	7	k__Bacteria; p__Firmicutes; c__Clostridia; o__Clostridiales; Unclassified; Unclassified; Unclassified
OTU242	34	4	k__Bacteria; p__Bacteroidota; c__Bacteroidia; o__Bacteroidales; f__Prevotellaceae; g__Prevotella; s__Prevotella_sp
OTU243	76	29	k__Bacteria; p__Firmicutes; c__Clostridia; o__Oscillospirales; f__Ruminococcaceae; g__Ruminococcus; s__Ruminococcus_bromii
OTU244	11	4	k__Bacteria; p__Firmicutes; c__Bacilli; Unclassified; Unclassified; Unclassified; Unclassified
OTU245	13	6	k__Bacteria; Unclassified; Unclassified; Unclassified; Unclassified; Unclassified; Unclassified
OTU246	44	38	k__Bacteria; p__Bacteroidota; c__Bacteroidia; o__Bacteroidales; f__Prevotellaceae; g__Prevotella; Unclassified
OTU247	9	5	k__Bacteria; p__Firmicutes; c__Clostridia; o__Oscillospirales; f__Ruminococcaceae; g__Ruminococcus; s__Ruminococcus_albus
OTU248	27	9	k__Bacteria; p__Bacteroidetes; c__Bacteroidia; o__Bacteroidales; f__Porphyromonadaceae; g__Gabonia; s__Gabonia_massiliensis
OTU249	17	15	k__Bacteria; p__Bacteroidota; c__Bacteroidia; Unclassified; Unclassified; Unclassified; Unclassified
OTU25	47	24	k__Bacteria; p__Firmicutes; c__Clostridia; o__Clostridiales; f__Hungateiclostridiaceae; Unclassified; Unclassified
OTU250	6	2	k__Bacteria; p__Bacteroidota; c__Bacteroidia; Unclassified; Unclassified; Unclassified; Unclassified
OTU251	28	28	k__Bacteria; p__Firmicutes; c__Clostridia; Unclassified; Unclassified; Unclassified; Unclassified
OTU252	10	4	k__Bacteria; p__Firmicutes; Unclassified; Unclassified; Unclassified; Unclassified; Unclassified
OTU253	82	52	k__Bacteria; Unclassified; Unclassified; Unclassified; Unclassified; Unclassified; Unclassified
OTU254	6	4	k__Bacteria; p__Spirochaetota; c__Spirochaetia; o__Spirochaetales; f__Spirochaetaceae; g__Treponema; s__Treponema_zioleckii
OTU255	18	2	k__Bacteria; p__Firmicutes; c__Clostridia; o__Clostridiales; Unclassified; Unclassified; Unclassified
OTU256	13	9	k__Bacteria; p__Bacteroidota; c__Bacteroidia; o__Bacteroidales; f__Prevotellaceae; g__Prevotella; s__Prevotella_sp
OTU257	12	15	k__Bacteria; p__Firmicutes; c__Bacilli; Unclassified; Unclassified; Unclassified; Unclassified
OTU258	14	6	k__Bacteria; p__Bacteroidota; c__Bacteroidia; o__Bacteroidales; f__Prevotellaceae; g__Prevotella; s__Prevotella_sp
OTU259	2	6	k__Bacteria; p__Firmicutes; c__Bacilli; Unclassified; Unclassified; Unclassified; Unclassified

OTU26	34	29	k__Bacteria; p__Bacteroidota; c__Bacteroidia; o__Bacteroidales; f__Rikenellaceae; Unclassified; Unclassified
OTU260	11	12	k__Bacteria; p__Firmicutes; c__Clostridia; o__Christensenellales; f__Christensenellaceae; g__Christensenella; Unclassified
OTU261	18	23	k__Bacteria; p__Firmicutes; c__Clostridia; Unclassified; Unclassified; Unclassified; Unclassified
OTU262	2	4	k__Bacteria; p__Firmicutes; c__Bacilli; Unclassified; Unclassified; Unclassified; Unclassified
OTU263	14	6	k__Bacteria; p__Firmicutes; c__Clostridia; Unclassified; Unclassified; Unclassified; Unclassified
OTU264	5	5	k__Bacteria; p__Firmicutes; c__Clostridia; Unclassified; Unclassified; Unclassified; Unclassified
OTU265	9	7	k__Bacteria; p__Firmicutes; c__Negativicutes; o__Veillonellales-Selenomonadales; f__Selenomonadaceae; g__Anaerovibrio; s__Anaerovibrio_lipolyticus
OTU266	29	18	k__Bacteria; p__Bacteroidota; c__Bacteroidia; o__Bacteroidales; f__Rikenellaceae; g__Bact; s__Bacteroidales_bacterium
OTU267	6	6	k__Bacteria; p__Firmicutes; c__Clostridia; o__Oscillospirales; f__Ruminococcaceae; g__Ruminococcus; s__Ruminococcus_flavefaciens
OTU268	30	45	k__Bacteria; p__Bacteroidota; c__Bacteroidia; o__Bacteroidales; f__Prevotellaceae; g__Prevotella; Unclassified
OTU269	12	6	k__Bacteria; p__Firmicutes; c__Clostridia; o__Oscillospirales; f__Oscillospiraceae; Unclassified; Unclassified
OTU27	19	13	k__Bacteria; p__Firmicutes; c__Bacilli; Unclassified; Unclassified; Unclassified; Unclassified
OTU270	9	10	k__Bacteria; p__Bacteroidota; c__Bacteroidia; o__Bacteroidales; f__Prevotellaceae; g__Prevotella; s__Prevotella_sp
OTU271	23	28	k__Bacteria; p__Firmicutes; c__Clostridia; Unclassified; Unclassified; Unclassified; Unclassified
OTU273	23	24	k__Bacteria; p__Bacteroidota; c__Bacteroidia; Unclassified; Unclassified; Unclassified; Unclassified
OTU274	14	7	k__Bacteria; Unclassified; Unclassified; Unclassified; Unclassified; Unclassified; Unclassified
OTU275	14	14	k__Bacteria; p__Bacteroidota; c__Bacteroidia; Unclassified; Unclassified; Unclassified; Unclassified
OTU276	4	5	k__Bacteria; p__Firmicutes; c__Bacilli; Unclassified; Unclassified; Unclassified; Unclassified
OTU278	9	5	k__Bacteria; Unclassified; Unclassified; Unclassified; Unclassified; Unclassified; Unclassified
OTU279	66	38	k__Bacteria; p__Verrucomicrobiota; Unclassified; Unclassified; Unclassified; Unclassified; Unclassified
OTU28	23	20	k__Bacteria; p__Firmicutes; c__Bacilli; Unclassified; Unclassified; Unclassified; Unclassified
OTU280	3	1	k__Bacteria; p__Firmicutes; Unclassified; Unclassified; Unclassified;

			Unclassified; Unclassified
OTU281	109	75	k__Bacteria; p__Verrucomicrobiota; Unclassified; Unclassified; Unclassified; Unclassified; Unclassified
OTU282	3	3	k__Bacteria; p__Firmicutes; c__Clostridia; Unclassified; Unclassified; Unclassified; Unclassified
OTU283	3	8	k__Bacteria; Unclassified; Unclassified; Unclassified; Unclassified; Unclassified; Unclassified
OTU284	17	25	k__Bacteria; p__Firmicutes; c__Clostridia; Unclassified; Unclassified; Unclassified; Unclassified
OTU285	36	27	k__Bacteria; p__Spirochaetota; c__Spirochaetia; o__Spirochaetales; f__Spirochaetaceae; g__Treponema; s__Treponema_bryantii
OTU286	10	2	k__Bacteria; p__Firmicutes; c__Bacilli; Unclassified; Unclassified; Unclassified; Unclassified
OTU287	79	33	k__Bacteria; p__Verrucomicrobiota; Unclassified; Unclassified; Unclassified; Unclassified; Unclassified
OTU288	8	11	k__Bacteria; Unclassified; Unclassified; Unclassified; Unclassified; Unclassified; Unclassified
OTU289	3	6	k__Bacteria; p__Bacteroidota; c__Bacteroidia; o__Bacteroidales; f__Prevotellaceae; g__Prevotella; s__Prevotella_sp
OTU29	105	53	k__Bacteria; p__Firmicutes; c__Bacilli; Unclassified; Unclassified; Unclassified; Unclassified
OTU291	10	9	k__Bacteria; p__Firmicutes; c__Clostridia; Unclassified; Unclassified; Unclassified; Unclassified
OTU292	10	5	k__Bacteria; p__Firmicutes; c__Clostridia; Unclassified; Unclassified; Unclassified; Unclassified
OTU293	38	41	k__Bacteria; p__Bacteroidota; c__Bacteroidia; o__Bacteroidales; f__Prevotellaceae; g__Prevotella; s__Prevotella_sp
OTU294	84	76	k__Bacteria; p__Firmicutes; c__Negativicutes; o__Veillonellales-Selenomonadales; f__Selenomonadaceae; Unclassified; Unclassified
OTU295	7	13	k__Bacteria; p__Firmicutes; c__Clostridia; Unclassified; Unclassified; Unclassified; Unclassified
OTU296	4	1	k__Bacteria; p__Firmicutes; c__Clostridia; Unclassified; Unclassified; Unclassified; Unclassified
OTU297	12	8	k__Bacteria; p__Patescibacteria; c__Saccharimonadia; o__Saccharimonadales; f__Saccharimonadaceae; g__Candidatus_Saccharimonas; s__Candidatus_Saccharimonas_aalborgensis
OTU299	4	3	k__Bacteria; p__Firmicutes; c__Clostridia; Unclassified; Unclassified; Unclassified; Unclassified
OTU3	92	43	k__Bacteria; p__Firmicutes; c__Clostridia; Unclassified; Unclassified; Unclassified; Unclassified
OTU30	54	51	k__Bacteria; p__Firmicutes; c__Clostridia; o__Oscillospirales; f__Ruminococcaceae; g__Ruminococcus; s__Ruminococcus_bromii
OTU301	1	8	k__Bacteria; p__Firmicutes; c__Clostridia; o__Oscillospirales;

			f__Oscillospiraceae; g__Oscillibacter; s__Oscillibacter_sp
OTU302	3	3	k__Bacteria; p__Firmicutes; Unclassified; Unclassified; Unclassified; Unclassified; Unclassified
OTU303	7	3	k__Bacteria; p__Bacteroidota; c__Bacteroidia; o__Bacteroidales; f__Prevotellaceae; g__Prevotella; s__Prevotella_sp
OTU305	3	8	k__Bacteria; p__Firmicutes; c__Bacilli; Unclassified; Unclassified; Unclassified; Unclassified
OTU306	5	3	k__Bacteria; p__Firmicutes; c__Bacilli; Unclassified; Unclassified; Unclassified; Unclassified
OTU307	42	74	k__Bacteria; p__Bacteroidota; c__Bacteroidia; o__Bacteroidales; f__Prevotellaceae; g__Prevotella; Unclassified
OTU308	9	10	k__Bacteria; p__Firmicutes; c__Clostridia; o__Oscillospirales; f__Oscillospiraceae; Unclassified; Unclassified
OTU31	43	29	k__Bacteria; p__Bacteroidota; c__Bacteroidia; o__Bacteroidales; f__Prevotellaceae; g__Prevotella; s__Prevotella_sp
OTU310	9	12	k__Bacteria; p__Firmicutes; c__Clostridia; o__Clostridiales; f__Clostridiales_Family_XIII._Incertae_Sedis; Unclassified; Unclassified
OTU311	28	20	k__Bacteria; p__Bacteroidota; c__Bacteroidia; o__Bacteroidales; f__Prevotellaceae; g__Prevotella; s__Prevotella_ruminicola
OTU312	0	8	k__Bacteria; p__Bacteroidota; c__Bacteroidia; o__Bacteroidales; f__Prevotellaceae; g__Prevotella; s__Prevotella_sp
OTU313	15	14	k__Bacteria; p__Firmicutes; c__Clostridia; Unclassified; Unclassified; Unclassified; Unclassified
OTU314	106	56	k__Bacteria; p__Bacteroidota; c__Bacteroidia; o__Bacteroidales; f__Prevotellaceae; g__Prevotella; s__Prevotella_sp
OTU316	5	3	k__Bacteria; p__Firmicutes; c__Clostridia; o__Oscillospirales; f__Ruminococcaceae; Unclassified; Unclassified
OTU317	9	2	k__Bacteria; p__Bacteroidota; c__Bacteroidia; o__Bacteroidales; Unclassified; Unclassified; Unclassified
OTU318	5	9	k__Bacteria; p__Firmicutes; c__Bacilli; o__Lactobacillales; f__Leuconostocaceae; g__Weissella; s__Weissella_confusa
OTU319	4	5	k__Bacteria; p__Spirochaetota; c__Spirochaetia; o__Spirochaetales; f__Spirochaetaceae; g__Treponema; s__Treponema_succinifaciens
OTU32	14	12	k__Bacteria; p__Firmicutes; c__Clostridia; o__Clostridiales; f__Hungateiclostridiaceae; Unclassified; Unclassified
OTU320	6	6	k__Bacteria; p__Firmicutes; c__Bacilli; o__Acholeplasmatales; f__Acholeplasmataceae; g__Anaeroplasma; s__Anaeroplasma_abactoclasticum
OTU321	22	22	k__Bacteria; p__Firmicutes; c__Clostridia; Unclassified; Unclassified; Unclassified; Unclassified
OTU323	23	16	k__Bacteria; p__Firmicutes; c__Clostridia; Unclassified; Unclassified; Unclassified; Unclassified
OTU324	7	7	k__Bacteria; Unclassified; Unclassified; Unclassified; Unclassified; Unclassified; Unclassified

OTU326	28	23	k__Bacteria; p__Firmicutes; c__Clostridia; o__Clostridiales; f__Hungateiclostridiaceae; Unclassified; Unclassified
OTU327	32	18	k__Bacteria; p__Bacteroidota; c__Bacteroidia; o__Bacteroidales; f__Prevotellaceae; g__Prevotella; Unclassified
OTU328	16	11	k__Bacteria; p__Firmicutes; c__Clostridia; Unclassified; Unclassified; Unclassified; Unclassified
OTU33	19	25	k__Bacteria; p__Bacteroidota; c__Bacteroidia; o__Bacteroidales; f__Prevotellaceae; g__Prevotella; Unclassified
OTU330	7	4	k__Bacteria; p__Proteobacteria; c__Deltaproteobacteria; o__Desulfuromonadales; Unclassified; Unclassified; Unclassified
OTU331	3	6	k__Bacteria; p__Firmicutes; Unclassified; Unclassified; Unclassified; Unclassified; Unclassified
OTU333	20	10	k__Bacteria; p__Verrucomicrobiota; Unclassified; Unclassified; Unclassified; Unclassified; Unclassified
OTU334	32	29	k__Bacteria; p__Bacteroidota; c__Bacteroidia; o__Bacteroidales; f__Rikenellaceae; g__Bact; s__Bacteroidales_bacterium
OTU335	8	9	k__Bacteria; p__Firmicutes; c__Clostridia; o__Christensenellales; f__Christensenellaceae; g__Christensenella; Unclassified
OTU336	12	10	k__Bacteria; p__Bacteroidota; c__Bacteroidia; o__Bacteroidales; Unclassified; Unclassified; Unclassified
OTU337	2	3	k__Bacteria; p__Bacteroidetes; c__Bacteroidia; o__Bacteroidales; f__Porphyromonadaceae; g__Gabonia; s__Gabonia_massiliensis
OTU338	3	3	k__Bacteria; p__Firmicutes; c__Clostridia; Unclassified; Unclassified; Unclassified; Unclassified
OTU339	17	16	k__Bacteria; p__Firmicutes; c__Clostridia; o__Clostridiales; Unclassified; Unclassified; Unclassified
OTU34	32	25	k__Bacteria; p__Firmicutes; c__Clostridia; o__Oscillospirales; f__Ruminococcaceae; g__Ruminococcus; s__Ruminococcus_sp
OTU340	12	15	k__Bacteria; p__Firmicutes; c__Clostridia; o__Eubacteriales; f__Eubacteriaceae; g__Eubacterium; s__Eubacterium_ruminantium
OTU341	2	7	k__Bacteria; p__Firmicutes; c__Clostridia; Unclassified; Unclassified; Unclassified; Unclassified
OTU342	73	51	k__Bacteria; p__Firmicutes; c__Clostridia; o__Oscillospirales; f__Oscillospiraceae; Unclassified; Unclassified
OTU344	6	10	k__Bacteria; p__Firmicutes; c__Clostridia; o__Clostridiales; f__Caloramatoraceae; g__Clostridium; s__Clostridium_methylpentosum
OTU346	10	13	k__Bacteria; p__Bacteroidota; c__Bacteroidia; o__Bacteroidales; f__Rikenellaceae; g__Bact; s__Bacteroidales_bacterium
OTU347	7	23	k__Bacteria; p__Bacteroidota; c__Bacteroidia; o__Bacteroidales; f__Prevotellaceae; g__Prevotella; s__Prevotella_sp
OTU349	5	2	k__Bacteria; p__Firmicutes; Unclassified; Unclassified; Unclassified; Unclassified; Unclassified
OTU35	12	5	k__Bacteria; p__Proteobacteria; Unclassified; Unclassified; Unclassified; Unclassified; Unclassified

OTU350	11	9	k__Bacteria; p__Bacteroidota; c__Bacteroidia; o__Bacteroidales; f__Prevotellaceae; g__Prevotella; s__Prevotella_sp
OTU352	10	8	k__Bacteria; p__Firmicutes; Unclassified; Unclassified; Unclassified; Unclassified; Unclassified
OTU353	13	12	k__Bacteria; p__Bacteroidota; c__Bacteroidia; o__Bacteroidales; f__Rikenellaceae; g__Bact; s__Bacteroidales_bacterium
OTU354	0	6	k__Bacteria; p__Firmicutes; Unclassified; Unclassified; Unclassified; Unclassified; Unclassified
OTU355	6	12	k__Bacteria; p__Bacteroidota; c__Bacteroidia; Unclassified; Unclassified; Unclassified; Unclassified
OTU356	45	31	k__Bacteria; p__Bacteroidota; c__Bacteroidia; Unclassified; Unclassified; Unclassified; Unclassified
OTU357	30	36	k__Bacteria; p__Bacteroidota; c__Bacteroidia; o__Bacteroidales; f__Prevotellaceae; g__Prevotella; s__Prevotella_sp
OTU358	10	3	k__Bacteria; p__Firmicutes; c__Clostridia; Unclassified; Unclassified; Unclassified; Unclassified
OTU359	7	10	k__Bacteria; Unclassified; Unclassified; Unclassified; Unclassified; Unclassified; Unclassified
OTU36	16	4	k__Bacteria; p__Firmicutes; c__Bacilli; o__Acholeplasmatales; f__Acholeplasmataceae; g__Anaeroplasma; Unclassified
OTU360	0	5	k__Bacteria; Unclassified; Unclassified; Unclassified; Unclassified; Unclassified; Unclassified
OTU361	31	27	k__Bacteria; p__Firmicutes; c__Clostridia; o__Oscillospirales; f__Oscillospiraceae; g__Sporobacter; s__Sporobacter_termitidis
OTU362	5	8	k__Bacteria; p__Firmicutes; c__Clostridia; o__Oscillospirales; f__Oscillospiraceae; Unclassified; Unclassified
OTU363	4	6	k__Bacteria; p__Bacteroidota; c__Bacteroidia; o__Bacteroidales; f__Rikenellaceae; g__Bact; s__Bacteroidales_bacterium
OTU364	6	6	k__Bacteria; p__Firmicutes; c__Clostridia; Unclassified; Unclassified; Unclassified; Unclassified
OTU367	11	14	k__Bacteria; p__Bacteroidota; c__Bacteroidia; o__Bacteroidales; f__Prevotellaceae; g__Prevotella; Unclassified
OTU368	9	6	k__Bacteria; p__Firmicutes; Unclassified; Unclassified; Unclassified; Unclassified; Unclassified
OTU37	150	105	k__Bacteria; Unclassified; Unclassified; Unclassified; Unclassified; Unclassified; Unclassified
OTU370	77	41	k__Bacteria; p__Bacteroidota; c__Bacteroidia; o__Bacteroidales; f__Prevotellaceae; g__Prevotella; Unclassified
OTU371	41	27	k__Bacteria; p__Bacteroidota; c__Bacteroidia; o__Bacteroidales; f__Prevotellaceae; g__Prevotella; s__Prevotella_sp
OTU373	4	7	k__Bacteria; p__Firmicutes; c__Bacilli; Unclassified; Unclassified; Unclassified; Unclassified
OTU374	2	3	k__Bacteria; p__Firmicutes; c__Clostridia; Unclassified; Unclassified; Unclassified; Unclassified

OTU375	5	2	k__Bacteria; p__Spirochaetota; c__Spirochaetia; o__Spirochaetales; f__Spirochaetaceae; g__Treponema; Unclassified
OTU377	2	8	k__Bacteria; p__Firmicutes; c__Clostridia; Unclassified; Unclassified; Unclassified; Unclassified
OTU379	2	5	k__Bacteria; p__Bacteroidota; c__Bacteroidia; o__Bacteroidales; f__Rikenellaceae; g__Bact; s__Bacteroidales_bacterium
OTU38	53	65	k__Bacteria; p__Verrucomicrobiota; Unclassified; Unclassified; Unclassified; Unclassified; Unclassified
OTU380	4	1	k__Bacteria; p__Firmicutes; c__Clostridia; Unclassified; Unclassified; Unclassified; Unclassified
OTU381	24	20	k__Bacteria; p__Bacteroidota; c__Bacteroidia; o__Bacteroidales; f__Prevotellaceae; g__Prevotella; s__Prevotella_sp
OTU382	40	39	k__Bacteria; p__Firmicutes; c__Bacilli; Unclassified; Unclassified; Unclassified; Unclassified
OTU383	5	5	k__Bacteria; Unclassified; Unclassified; Unclassified; Unclassified; Unclassified; Unclassified
OTU384	7	12	k__Bacteria; Unclassified; Unclassified; Unclassified; Unclassified; Unclassified; Unclassified
OTU386	36	36	k__Bacteria; p__Bacteroidota; c__Bacteroidia; o__Bacteroidales; f__Prevotellaceae; g__Prevotella; s__Prevotella_ruminicola
OTU388	3	8	k__Bacteria; p__Firmicutes; c__Clostridia; o__Oscillospirales; f__Ruminococcaceae; g__Ruminococcus; s__Ruminococcus_bromii
OTU389	6	3	k__Bacteria; p__Firmicutes; c__Clostridia; o__Oscillospirales; f__Ruminococcaceae; g__Ruminococcus; s__Ruminococcus_flavefaciens
OTU39	19	19	k__Bacteria; p__Firmicutes; c__Clostridia; Unclassified; Unclassified; Unclassified; Unclassified
OTU390	2	6	k__Bacteria; p__Firmicutes; c__Clostridia; o__Clostridiales; f__Clostridiales_Family_XIII._Incertae_Sedis; Unclassified; Unclassified
OTU392	36	22	k__Bacteria; p__Bacteroidota; c__Bacteroidia; o__Bacteroidales; Unclassified; Unclassified; Unclassified
OTU393	4	11	k__Bacteria; Unclassified; Unclassified; Unclassified; Unclassified; Unclassified; Unclassified
OTU394	4	5	k__Bacteria; p__Firmicutes; c__Bacilli; Unclassified; Unclassified; Unclassified; Unclassified
OTU395	14	11	k__Bacteria; Unclassified; Unclassified; Unclassified; Unclassified; Unclassified; Unclassified
OTU397	14	8	k__Bacteria; p__Fibrobacterota; c__Fibrobacteria; o__Fibrobacterales; f__Fibrobacteraceae; g__Fibrobacter; s__Fibrobacter_sp
OTU398	8	11	k__Bacteria; Unclassified; Unclassified; Unclassified; Unclassified; Unclassified; Unclassified
OTU399	31	35	k__Bacteria; p__Bacteroidota; c__Bacteroidia; o__Bacteroidales; f__Prevotellaceae; g__Prevotella; s__Prevotella_sp
OTU4	377	208	k__Bacteria; p__Bacteroidota; c__Bacteroidia; o__Bacteroidales; f__Prevotellaceae; g__Prevotella; Unclassified

OTU40	15	22	k__Bacteria; p__Firmicutes; c__Clostridia; o__Lachnospirales; f__Lachnospiraceae; g__Butyrivibrio; s__Butyrivibrio_hungatei
OTU400	3	3	k__Bacteria; p__Firmicutes; c__Clostridia; Unclassified; Unclassified; Unclassified; Unclassified
OTU402	15	18	k__Bacteria; p__Bacteroidota; c__Bacteroidia; Unclassified; Unclassified; Unclassified; Unclassified
OTU403	6	11	k__Bacteria; p__Bacteroidota; c__Bacteroidia; o__Bacteroidales; f__Prevotellaceae; g__Prevotella; Unclassified
OTU404	27	37	k__Bacteria; p__Firmicutes; c__Bacilli; Unclassified; Unclassified; Unclassified; Unclassified
OTU405	9	7	k__Bacteria; Unclassified; Unclassified; Unclassified; Unclassified; Unclassified; Unclassified
OTU406	4	11	k__Bacteria; p__Firmicutes; c__Bacilli; Unclassified; Unclassified; Unclassified; Unclassified
OTU407	5	2	k__Bacteria; p__Firmicutes; Unclassified; Unclassified; Unclassified; Unclassified; Unclassified
OTU408	27	2	k__Bacteria; p__Firmicutes; c__Clostridia; Unclassified; Unclassified; Unclassified; Unclassified
OTU41	10	12	k__Bacteria; Unclassified; Unclassified; Unclassified; Unclassified; Unclassified; Unclassified
OTU410	6	4	k__Bacteria; p__Firmicutes; c__Bacilli; Unclassified; Unclassified; Unclassified; Unclassified
OTU411	6	10	k__Bacteria; p__Bacteroidetes; c__Bacteroidia; o__Bacteroidales; f__Porphyromonadaceae; g__Gabonia; s__Gabonia_massiliensis
OTU412	5	7	k__Bacteria; p__Bacteroidota; c__Bacteroidia; Unclassified; Unclassified; Unclassified; Unclassified
OTU413	14	6	k__Bacteria; Unclassified; Unclassified; Unclassified; Unclassified; Unclassified; Unclassified
OTU414	2	4	k__Bacteria; p__Firmicutes; c__Clostridia; o__Clostridiales; f__Clostridiales_Family_XIII._Incertae_Sedis; Unclassified; Unclassified
OTU415	4	3	k__Bacteria; p__Firmicutes; c__Clostridia; o__Lachnospirales; f__Lachnospiraceae; g__Roseburia; s__Roseburia_sp
OTU417	17	12	k__Bacteria; p__Bacteroidota; c__Bacteroidia; Unclassified; Unclassified; Unclassified; Unclassified
OTU418	4	11	k__Bacteria; p__Proteobacteria; c__Deltaproteobacteria; o__Desulfuromonadales; Unclassified; Unclassified; Unclassified
OTU419	4	3	k__Bacteria; p__Bacteroidota; c__Bacteroidia; Unclassified; Unclassified; Unclassified; Unclassified
OTU42	24	27	k__Bacteria; p__Planctomycetota; c__Planctomycetes; o__Pirellulales; f__Pirellulaceae; Unclassified; Unclassified
OTU420	4	9	k__Bacteria; p__Spirochaetota; c__Spirochaetia; o__Spirochaetales; f__Spirochaetaceae; g__Treponema; s__Treponema_bryantii
OTU421	3	10	k__Bacteria; p__Bacteroidota; c__Bacteroidia; o__Bacteroidales; f__Prevotellaceae; g__Prevotella; Unclassified

OTU422	2	7	k__Bacteria; p__Firmicutes; c__Clostridia; o__Lachnospirales; f__Lachnospiraceae; g__Butyrivibrio; s__Butyrivibrio_sp
OTU423	0	5	k__Bacteria; p__Bacteroidota; c__Bacteroidia; o__Bacteroidales; f__Rikenellaceae; g__Bact; s__Bacteroidales_bacterium
OTU424	3	2	k__Bacteria; p__Firmicutes; Unclassified; Unclassified; Unclassified; Unclassified; Unclassified
OTU425	7	3	k__Bacteria; p__Patescibacteria; c__Saccharimonadia; o__Saccharimonadales; f__Saccharimonadaceae; g__Candidatus_Saccharimonas; s__Candidatus_Saccharimonas_aalborgensis
OTU426	32	23	k__Bacteria; p__Bacteroidota; c__Bacteroidia; o__Bacteroidales; Unclassified; Unclassified; Unclassified
OTU427	9	4	k__Bacteria; p__Firmicutes; c__Clostridia; Unclassified; Unclassified; Unclassified; Unclassified
OTU428	4	11	k__Bacteria; p__Firmicutes; c__Clostridia; Unclassified; Unclassified; Unclassified; Unclassified
OTU429	25	17	k__Bacteria; Unclassified; Unclassified; Unclassified; Unclassified; Unclassified; Unclassified
OTU43	12	14	k__Bacteria; p__Firmicutes; c__Clostridia; o__Lachnospirales; f__Lachnospiraceae; g__Lachnospira; s__Lachnospira_pectinoschiza
OTU432	4	8	k__Bacteria; Unclassified; Unclassified; Unclassified; Unclassified; Unclassified; Unclassified
OTU433	4	5	k__Bacteria; p__Firmicutes; c__Bacilli; Unclassified; Unclassified; Unclassified; Unclassified
OTU434	2	8	k__Bacteria; p__Firmicutes; c__Clostridia; Unclassified; Unclassified; Unclassified; Unclassified
OTU435	19	17	k__Bacteria; p__Bacteroidota; c__Bacteroidia; o__Bacteroidales; f__Prevotellaceae; g__Prevotella; Unclassified
OTU436	5	5	k__Bacteria; p__Firmicutes; c__Clostridia; Unclassified; Unclassified; Unclassified; Unclassified
OTU437	37	50	k__Bacteria; p__Bacteroidota; c__Bacteroidia; o__Bacteroidales; f__Prevotellaceae; g__Prevotella; Unclassified
OTU438	21	15	k__Bacteria; p__Firmicutes; c__Negativicutes; o__Veillonellales-Selenomonadales; f__Selenomonadaceae; g__Anaerovibrio; s__Anaerovibrio_lipolyticus
OTU439	3	6	k__Bacteria; p__Verrucomicrobiota; Unclassified; Unclassified; Unclassified; Unclassified; Unclassified
OTU44	4	4	k__Bacteria; p__Firmicutes; c__Clostridia; Unclassified; Unclassified; Unclassified; Unclassified
OTU441	2	5	k__Bacteria; p__Desulfobacterota; c__Desulfovibrionia; o__Desulfovibrionales; f__Desulfovibrionaceae; g__Desulfovibrio; s__Desulfovibrio_sp
OTU442	10	10	k__Bacteria; Unclassified; Unclassified; Unclassified; Unclassified; Unclassified; Unclassified

OTU444	3	3	k__Bacteria; p__Planctomycetota; c__Planctomycetes; o__Pirellulales; f__Pirellulaceae; Unclassified; Unclassified
OTU445	6	3	k__Bacteria; p__Firmicutes; c__Clostridia; Unclassified; Unclassified; Unclassified; Unclassified
OTU446	3	2	k__Bacteria; p__Bacteroidota; c__Bacteroidia; o__Bacteroidales; f__Prevotellaceae; g__Prevotella; Unclassified
OTU447	13	11	k__Bacteria; p__Bacteroidota; c__Bacteroidia; o__Bacteroidales; f__Prevotellaceae; g__Prevotella; Unclassified
OTU448	6	4	k__Bacteria; p__Firmicutes; c__Clostridia; o__Clostridiales; f__Hungateiclostridiaceae; Unclassified; Unclassified
OTU449	7	4	k__Bacteria; p__Firmicutes; Unclassified; Unclassified; Unclassified; Unclassified; Unclassified
OTU45	21	16	k__Bacteria; p__Cyanobacteria; Unclassified; Unclassified; Unclassified; Unclassified; Unclassified
OTU451	15	21	k__Bacteria; p__Firmicutes; c__Clostridia; o__Clostridiales; f__Hungateiclostridiaceae; Unclassified; Unclassified
OTU452	6	7	k__Bacteria; p__Firmicutes; Unclassified; Unclassified; Unclassified; Unclassified; Unclassified
OTU453	6	2	k__Bacteria; p__Elusimicrobiota; c__Endomicrobia; o__Endomicrobiales; f__Endomicrobiaceae; g__Endomicrobium; Unclassified
OTU454	1	4	k__Bacteria; p__Proteobacteria; c__Alphaproteobacteria; Unclassified; Unclassified; Unclassified; Unclassified
OTU455	13	10	k__Bacteria; p__Bacteroidetes; c__Bacteroidia; o__Bacteroidales; f__Porphyromonadaceae; g__Gabonia; s__Gabonia_massiliensis
OTU456	9	9	k__Bacteria; p__Firmicutes; c__Clostridia; o__Eubacteriales; f__Eubacteriaceae; g__Eubacterium; s__Eubacterium_ruminantium
OTU458	7	4	k__Bacteria; p__Firmicutes; c__Clostridia; o__Lachnospirales; f__Lachnospiraceae; Unclassified; Unclassified
OTU459	18	22	k__Bacteria; p__Firmicutes; Unclassified; Unclassified; Unclassified; Unclassified; Unclassified
OTU46	46	17	k__Bacteria; p__Bacteroidota; c__Bacteroidia; o__Bacteroidales; f__Rikenellaceae; g__Anaerocella; s__Anaerocella_delicata
OTU461	21	13	k__Bacteria; Unclassified; Unclassified; Unclassified; Unclassified; Unclassified; Unclassified
OTU462	9	11	k__Bacteria; Unclassified; Unclassified; Unclassified; Unclassified; Unclassified; Unclassified
OTU463	9	4	k__Bacteria; p__Firmicutes; c__Bacilli; Unclassified; Unclassified; Unclassified; Unclassified
OTU464	5	1	k__Bacteria; p__Firmicutes; c__Bacilli; Unclassified; Unclassified; Unclassified; Unclassified
OTU465	8	25	k__Bacteria; Unclassified; Unclassified; Unclassified; Unclassified; Unclassified; Unclassified
OTU466	20	21	k__Bacteria; p__Bacteroidota; c__Bacteroidia; o__Bacteroidales; f__Prevotellaceae; g__Prevotella; Unclassified

OTU467	3	5	k__Bacteria; p__Firmicutes; c__Clostridia; o__Clostridiales; f__Hungateiclostridiaceae; Unclassified; Unclassified
OTU468	2	6	k__Bacteria; Unclassified; Unclassified; Unclassified; Unclassified; Unclassified; Unclassified
OTU469	4	6	k__Bacteria; p__Firmicutes; c__Clostridia; o__Oscillospirales; f__Oscillospiraceae; Unclassified; Unclassified
OTU47	6	3	k__Bacteria; p__Firmicutes; Unclassified; Unclassified; Unclassified; Unclassified; Unclassified
OTU473	2	2	k__Bacteria; Unclassified; Unclassified; Unclassified; Unclassified; Unclassified; Unclassified
OTU474	5	4	k__Bacteria; p__Bacteroidota; c__Bacteroidia; o__Bacteroidales; f__Rikenellaceae; g__Bact; s__Bacteroidales_bacterium
OTU475	15	10	k__Bacteria; Unclassified; Unclassified; Unclassified; Unclassified; Unclassified; Unclassified
OTU476	3	6	k__Bacteria; p__Firmicutes; c__Clostridia; o__Oscillospirales; f__Ruminococcaceae; Unclassified; Unclassified
OTU478	4	9	k__Bacteria; p__Bacteroidota; c__Bacteroidia; o__Bacteroidales; Unclassified; Unclassified; Unclassified
OTU479	7	10	k__Bacteria; p__Firmicutes; c__Clostridia; o__Christensenellales; f__Christensenellaceae; g__Christensenella; Unclassified
OTU48	21	2	k__Bacteria; p__Firmicutes; Unclassified; Unclassified; Unclassified; Unclassified; Unclassified
OTU482	8	3	k__Bacteria; p__Firmicutes; c__Bacilli; Unclassified; Unclassified; Unclassified; Unclassified
OTU484	11	6	k__Bacteria; p__Firmicutes; c__Clostridia; Unclassified; Unclassified; Unclassified; Unclassified
OTU485	8	1	k__Bacteria; p__Firmicutes; c__Clostridia; o__Clostridiales; f__Hungateiclostridiaceae; Unclassified; Unclassified
OTU486	8	7	k__Bacteria; p__Firmicutes; c__Clostridia; o__Lachnospirales; f__Lachnospiraceae; g__Butyrivibrio; s__Butyrivibrio_sp
OTU487	4	5	k__Bacteria; p__Bacteroidota; c__Bacteroidia; o__Bacteroidales; f__Prevotellaceae; g__Prevotella; s__Prevotella_sp
OTU489	5	10	k__Bacteria; p__Firmicutes; c__Clostridia; Unclassified; Unclassified; Unclassified; Unclassified
OTU49	26	12	k__Bacteria; Unclassified; Unclassified; Unclassified; Unclassified; Unclassified; Unclassified
OTU491	42	31	k__Bacteria; Unclassified; Unclassified; Unclassified; Unclassified; Unclassified; Unclassified
OTU492	3	3	k__Bacteria; p__Desulfobacterota; c__Desulfovibrionia; o__Desulfovibrionales; f__Desulfovibrionaceae; g__Bilophila; s__Bilophila_sp
OTU493	4	5	k__Bacteria; p__Firmicutes; c__Bacilli; o__Erysipelotrichales; f__Erysipelotrichaceae; g__Solobacterium; s__Solobacterium_sp
OTU494	11	6	k__Bacteria; p__Spirochaetota; c__Spirochaetia; o__Spirochaetales;

			f__Spirochaetaceae; g__Treponema; s__Treponema_zioleckii
OTU495	3	3	k__Bacteria; p__Firmicutes; c__Bacilli; o__Erysipelotrichales; f__Erysipelotrichaceae; Unclassified; Unclassified
OTU496	78	89	k__Bacteria; p__Firmicutes; c__Negativicutes; o__Veillonellales-Selenomonadales; f__Selenomonadaceae; g__Selenomonas; s__Selenomonas_ruminantium
OTU498	2	9	k__Bacteria; p__Firmicutes; c__Clostridia; o__Clostridiales; f__Hungateiclostridiaceae; Unclassified; Unclassified
OTU499	1	6	k__Bacteria; p__Actinobacteriota; c__Actinobacteria; o__Bifidobacteriales; f__Bifidobacteriaceae; g__Bifidobacterium; s__Bifidobacterium_sp
OTU5	135	90	k__Bacteria; p__Planctomycetota; c__Planctomycetes; o__Pirellulales; f__Pirellulaceae; Unclassified; Unclassified
OTU50	24	19	k__Bacteria; p__Firmicutes; c__Clostridia; o__Clostridiales; f__Hungateiclostridiaceae; Unclassified; Unclassified
OTU500	6	3	k__Bacteria; p__Firmicutes; c__Clostridia; o__Oscillospirales; f__Ruminococcaceae; Unclassified; Unclassified
OTU501	7	12	k__Bacteria; p__Verrucomicrobiota; Unclassified; Unclassified; Unclassified; Unclassified; Unclassified
OTU502	8	10	k__Bacteria; p__Firmicutes; c__Clostridia; Unclassified; Unclassified; Unclassified; Unclassified
OTU504	22	23	k__Bacteria; p__Bacteroidetes; c__Bacteroidia; o__Bacteroidales; f__Prevotellaceae; Unclassified; Unclassified
OTU505	9	3	k__Bacteria; p__Firmicutes; c__Clostridia; Unclassified; Unclassified; Unclassified; Unclassified
OTU506	11	11	k__Bacteria; p__Bacteroidota; c__Bacteroidia; o__Bacteroidales; f__Prevotellaceae; g__Prevotella; s__Prevotella_sp
OTU507	0	8	k__Bacteria; p__Bacteroidota; c__Bacteroidia; o__Bacteroidales; f__Prevotellaceae; g__Prevotella; s__Prevotella_ruminicola
OTU509	2	1	k__Bacteria; p__Firmicutes; c__Clostridia; Unclassified; Unclassified; Unclassified; Unclassified
OTU51	3	6	k__Bacteria; p__Firmicutes; c__Clostridia; o__Clostridiales; f__Hungateiclostridiaceae; g__Saccharofermentans; s__Saccharofermentans_acetigenes
OTU510	5	5	k__Bacteria; p__Firmicutes; c__Clostridia; o__Eubacteriales; f__Eubacteriaceae; g__Eubacterium; s__Eubacterium_ruminantium
OTU511	21	17	k__Bacteria; p__Firmicutes; c__Clostridia; o__Oscillospirales; f__Ruminococcaceae; g__Ruminococcus; s__Ruminococcus_bromii
OTU512	39	35	k__Bacteria; p__Bacteroidota; c__Bacteroidia; o__Bacteroidales; f__Prevotellaceae; g__Prevotella; Unclassified
OTU513	26	13	k__Bacteria; p__Bacteroidota; c__Bacteroidia; o__Bacteroidales; f__Prevotellaceae; g__Prevotella; Unclassified
OTU515	6	4	k__Bacteria; p__Firmicutes; c__Clostridia; o__Oscillospirales; f__Ruminococcaceae; g__Ruminococcus; s__Ruminococcus_sp
OTU516	5	3	k__Bacteria; p__Firmicutes; c__Clostridia; o__Oscillospirales;

			f__Ruminococcaceae; Unclassified; Unclassified
OTU518	6	3	k__Bacteria; p__Bacteroidota; c__Bacteroidia; o__Bacteroidales; f__Prevotellaceae; g__Prevotella; s__Prevotella_sp
OTU519	9	5	k__Bacteria; p__Firmicutes; c__Clostridia; o__Eubacteriales; f__Eubacteriaceae; g__Eubacterium; s__Eubacterium_siraeum
OTU52	10	10	k__Bacteria; p__Proteobacteria; c__Gammaproteobacteria; o__Aeromonadales; f__Succinivibrionaceae; g__Ruminobacter; Unclassified
OTU520	43	22	k__Bacteria; p__Bacteroidota; c__Bacteroidia; o__Bacteroidales; f__Prevotellaceae; g__Prevotella; s__Prevotella_sp
OTU522	18	3	k__Bacteria; p__Firmicutes; Unclassified; Unclassified; Unclassified; Unclassified; Unclassified
OTU523	7	3	k__Bacteria; p__Firmicutes; c__Clostridia; o__Clostridiales; Unclassified; Unclassified; Unclassified
OTU525	14	0	k__Bacteria; p__Firmicutes; Unclassified; Unclassified; Unclassified; Unclassified; Unclassified
OTU526	6	3	k__Bacteria; p__Firmicutes; c__Clostridia; o__Clostridiales; f__Hungateiclostridiaceae; Unclassified; Unclassified
OTU527	5	5	k__Bacteria; p__Firmicutes; c__Clostridia; o__Eubacteriales; f__Eubacteriaceae; g__Eubacterium; s__Eubacterium_uniforme
OTU528	6	1	k__Bacteria; p__Proteobacteria; c__Alphaproteobacteria; o__Rhodospirillales; f__Terasakiellaceae; g__Aestuariispira; s__Aestuariispira_insulae
OTU529	10	5	k__Bacteria; Unclassified; Unclassified; Unclassified; Unclassified; Unclassified; Unclassified
OTU53	5	3	k__Bacteria; p__Planctomycetota; c__Planctomycetes; o__Pirellulales; f__Pirellulaceae; Unclassified; Unclassified
OTU530	15	5	k__Bacteria; p__Firmicutes; c__Bacilli; o__Acholeplasmatales; f__Acholeplasmataceae; g__Anaeroplasma; s__Anaeroplasma_varium
OTU531	14	16	k__Bacteria; p__Bacteroidota; c__Bacteroidia; o__Bacteroidales; f__Prevotellaceae; g__Prevotella; Unclassified
OTU532	0	3	k__Bacteria; p__Bacteroidota; c__Bacteroidia; Unclassified; Unclassified; Unclassified; Unclassified
OTU533	7	6	k__Bacteria; p__Firmicutes; c__Clostridia; Unclassified; Unclassified; Unclassified; Unclassified
OTU534	12	28	k__Bacteria; p__Proteobacteria; c__Gammaproteobacteria; o__Enterobacterales; f__Enterobacteriaceae; g__Klebsiella; s__Klebsiella_pneumoniae
OTU535	66	88	k__Bacteria; p__Bacteroidota; c__Bacteroidia; o__Bacteroidales; f__Prevotellaceae; g__Prevotella; Unclassified
OTU536	4	3	k__Bacteria; p__Bacteroidota; c__Bacteroidia; o__Bacteroidales; f__Prevotellaceae; g__Prevotella; Unclassified
OTU537	6	3	k__Bacteria; p__Firmicutes; c__Bacilli; Unclassified; Unclassified; Unclassified; Unclassified

OTU538	6	10	k__Bacteria; p__Firmicutes; c__Clostridia; o__Clostridiales; Unclassified; Unclassified; Unclassified
OTU539	76	37	k__Bacteria; p__Bacteroidota; c__Bacteroidia; o__Bacteroidales; f__Prevotellaceae; g__Prevotella; s__Prevotella_ruminicola
OTU54	12	29	k__Bacteria; p__Firmicutes; c__Clostridia; Unclassified; Unclassified; Unclassified; Unclassified
OTU541	4	2	k__Bacteria; p__Firmicutes; c__Clostridia; o__Eubacteriales; f__Eubacteriaceae; g__Eubacterium; s__Eubacterium_ventriosum
OTU542	19	17	k__Bacteria; p__Bacteroidota; c__Bacteroidia; o__Bacteroidales; Unclassified; Unclassified; Unclassified
OTU543	7	0	k__Bacteria; p__Firmicutes; c__Bacilli; Unclassified; Unclassified; Unclassified; Unclassified
OTU544	4	0	k__Bacteria; p__Verrucomicrobiota; Unclassified; Unclassified; Unclassified; Unclassified; Unclassified
OTU545	4	3	k__Bacteria; p__Firmicutes; Unclassified; Unclassified; Unclassified; Unclassified; Unclassified
OTU546	35	6	k__Bacteria; p__Firmicutes; c__Clostridia; o__Clostridiales; Unclassified; Unclassified; Unclassified
OTU547	6	3	k__Bacteria; p__Firmicutes; c__Bacilli; Unclassified; Unclassified; Unclassified; Unclassified
OTU548	9	5	k__Bacteria; Unclassified; Unclassified; Unclassified; Unclassified; Unclassified; Unclassified
OTU55	17	11	k__Bacteria; p__Firmicutes; c__Clostridia; o__Clostridiales; f__Hungateiclostridiaceae; Unclassified; Unclassified
OTU551	10	4	k__Bacteria; p__Bacteroidota; c__Bacteroidia; o__Bacteroidales; f__Rikenellaceae; g__Bact; s__Bacteroidales_bacterium
OTU552	6	0	k__Bacteria; p__Firmicutes; c__Clostridia; Unclassified; Unclassified; Unclassified; Unclassified
OTU556	7	0	k__Bacteria; p__Firmicutes; c__Clostridia; o__Clostridiales; f__Hungateiclostridiaceae; Unclassified; Unclassified
OTU557	5	2	k__Bacteria; p__Firmicutes; c__Bacilli; Unclassified; Unclassified; Unclassified; Unclassified
OTU558	6	2	k__Bacteria; p__Firmicutes; c__Clostridia; o__Lachnospirales; f__Lachnospiraceae; g__Pseudobutyrivibrio; Unclassified
OTU559	16	16	k__Bacteria; p__Firmicutes; Unclassified; Unclassified; Unclassified; Unclassified; Unclassified
OTU56	56	71	k__Bacteria; p__Firmicutes; c__Clostridia; o__Lachnospirales; f__Lachnospiraceae; g__Pseudobutyrivibrio; s__Pseudobutyrivibrio_sp
OTU560	8	5	k__Bacteria; p__Firmicutes; c__Bacilli; Unclassified; Unclassified; Unclassified; Unclassified
OTU561	69	67	k__Bacteria; p__Bacteroidota; c__Bacteroidia; o__Bacteroidales; f__Prevotellaceae; g__Prevotella; s__Prevotella_sp
OTU562	119	75	k__Bacteria; p__Bacteroidota; c__Bacteroidia; o__Bacteroidales; Unclassified; Unclassified; Unclassified

OTU564	24	18	k__Bacteria; Unclassified; Unclassified; Unclassified; Unclassified; Unclassified; Unclassified
OTU565	25	30	k__Bacteria; p__Bacteroidota; c__Bacteroidia; o__Bacteroidales; f__Rikenellaceae; g__Bact; s__Bacteroidales_bacterium
OTU567	8	1	k__Bacteria; Unclassified; Unclassified; Unclassified; Unclassified; Unclassified; Unclassified
OTU569	23	28	k__Bacteria; p__Bacteroidota; c__Bacteroidia; o__Bacteroidales; f__Prevotellaceae; g__Prevotella; Unclassified
OTU57	32	35	k__Bacteria; p__Firmicutes; c__Bacilli; Unclassified; Unclassified; Unclassified; Unclassified
OTU570	19	7	k__Bacteria; p__Bacteroidetes; c__Bacteroidia; o__Bacteroidales; f__Prevotellaceae; g__Marseilla; s__Marseilla_massiliensis
OTU571	3	6	k__Bacteria; p__Firmicutes; c__Clostridia; Unclassified; Unclassified; Unclassified; Unclassified
OTU572	24	11	k__Bacteria; Unclassified; Unclassified; Unclassified; Unclassified; Unclassified; Unclassified
OTU573	3	2	k__Bacteria; p__Firmicutes; c__Bacilli; Unclassified; Unclassified; Unclassified; Unclassified
OTU574	1	3	k__Bacteria; p__Bacteroidota; c__Bacteroidia; o__Bacteroidales; Unclassified; Unclassified; Unclassified
OTU575	6	4	k__Bacteria; p__Bacteroidota; c__Bacteroidia; o__Bacteroidales; Unclassified; Unclassified; Unclassified
OTU576	4	3	k__Bacteria; Unclassified; Unclassified; Unclassified; Unclassified; Unclassified; Unclassified
OTU577	4	2	k__Bacteria; p__Proteobacteria; Unclassified; Unclassified; Unclassified; Unclassified; Unclassified
OTU578	13	13	k__Bacteria; p__Firmicutes; c__Clostridia; o__Christensenellales; f__Christensenellaceae; g__Christensenella; s__Christensenella_sp
OTU579	8	2	k__Bacteria; p__Proteobacteria; c__Alphaproteobacteria; o__Rhodospirillales; f__Terasakiellaceae; g__Aestuariispira; s__Aestuariispira_insulae
OTU58	11	5	k__Bacteria; p__Firmicutes; c__Bacilli; Unclassified; Unclassified; Unclassified; Unclassified
OTU580	15	11	k__Bacteria; p__Firmicutes; c__Clostridia; o__Oscillospirales; f__Ruminococcaceae; g__Ruminococcus; s__Ruminococcus_sp
OTU581	17	16	k__Bacteria; p__Bacteroidota; c__Bacteroidia; o__Bacteroidales; f__Prevotellaceae; g__Prevotella; s__Prevotella_sp
OTU582	6	0	k__Bacteria; p__Firmicutes; Unclassified; Unclassified; Unclassified; Unclassified; Unclassified
OTU583	7	1	k__Bacteria; p__Firmicutes; c__Clostridia; Unclassified; Unclassified; Unclassified; Unclassified
OTU584	5	2	k__Bacteria; p__Firmicutes; c__Bacilli; Unclassified; Unclassified; Unclassified; Unclassified
OTU586	6	5	k__Bacteria; p__Bacteroidota; c__Bacteroidia; o__Bacteroidales;

			f__Prevotellaceae; g__Prevotella; Unclassified
OTU588	12	8	k__Bacteria; p__Firmicutes; c__Clostridia; Unclassified; Unclassified; Unclassified; Unclassified
OTU59	4	10	k__Bacteria; p__Firmicutes; c__Clostridia; o__Eubacteriales; f__Eubacteriaceae; Unclassified; Unclassified
OTU590	20	15	k__Bacteria; Unclassified; Unclassified; Unclassified; Unclassified; Unclassified; Unclassified
OTU591	8	2	k__Bacteria; p__Firmicutes; c__Bacilli; Unclassified; Unclassified; Unclassified; Unclassified
OTU592	6	4	k__Bacteria; p__Firmicutes; c__Clostridia; o__Clostridiales; Unclassified; Unclassified; Unclassified
OTU593	2	2	k__Bacteria; p__Firmicutes; c__Clostridia; o__Oscillospirales; f__Ruminococcaceae; g__Ruminococcus; s__Ruminococcus_flavefaciens
OTU594	9	6	k__Bacteria; p__Firmicutes; c__Clostridia; o__Oscillospirales; f__Oscillospiraceae; Unclassified; Unclassified
OTU596	5	5	k__Bacteria; p__Firmicutes; c__Clostridia; o__Oscillospirales; f__Oscillospiraceae; Unclassified; Unclassified
OTU597	7	6	k__Bacteria; p__Bacteroidota; c__Bacteroidia; o__Bacteroidales; f__Prevotellaceae; g__Prevotella; Unclassified
OTU599	5	4	k__Bacteria; p__Bacteroidota; c__Bacteroidia; o__Bacteroidales; f__Prevotellaceae; g__Prevotella; s__Prevotella_sp
OTU6	564	900	k__Bacteria; p__Proteobacteria; c__Gammaproteobacteria; Unclassified; Unclassified; Unclassified; Unclassified
OTU60	26	20	k__Bacteria; p__Firmicutes; c__Clostridia; o__Oscillospirales; f__Oscillospiraceae; g__Oscillibacter; s__Oscillibacter_sp
OTU600	9	3	k__Bacteria; p__Firmicutes; c__Bacilli; o__Acholeplasmatales; f__Acholeplasmataceae; g__Anaeroplasma; s__Anaeroplasma_abactoclasticum
OTU603	7	3	k__Bacteria; p__Firmicutes; Unclassified; Unclassified; Unclassified; Unclassified; Unclassified
OTU604	10	6	k__Bacteria; p__Firmicutes; c__Clostridia; Unclassified; Unclassified; Unclassified; Unclassified
OTU605	3	2	k__Bacteria; p__Firmicutes; c__Clostridia; Unclassified; Unclassified; Unclassified; Unclassified
OTU61	15	13	k__Bacteria; p__Bacteroidota; c__Bacteroidia; o__Bacteroidales; f__Rikenellaceae; g__Bact; s__Bacteroidales_bacterium
OTU62	12	12	k__Bacteria; p__Firmicutes; c__Bacilli; Unclassified; Unclassified; Unclassified; Unclassified
OTU63	28	11	k__Bacteria; Unclassified; Unclassified; Unclassified; Unclassified; Unclassified; Unclassified
OTU64	32	24	k__Bacteria; p__Verrucomicrobiota; c__Verrucomicrobiae; Unclassified; Unclassified; Unclassified; Unclassified
OTU65	8	2	k__Bacteria; p__Firmicutes; Unclassified; Unclassified; Unclassified; Unclassified; Unclassified

OTU66	9	18	k__Bacteria; p__Firmicutes; c__Clostridia; Unclassified; Unclassified; Unclassified; Unclassified
OTU67	3	12	k__Bacteria; p__Firmicutes; Unclassified; Unclassified; Unclassified; Unclassified; Unclassified
OTU68	4	2	k__Bacteria; p__Firmicutes; c__Bacilli; o__Erysipelotrichales; f__Erysipelotrichaceae; Unclassified; Unclassified
OTU69	12	12	k__Bacteria; Unclassified; Unclassified; Unclassified; Unclassified; Unclassified; Unclassified
OTU7	384	609	k__Bacteria; p__Firmicutes; c__Negativicutes; o__Acidaminococcales; f__Acidaminococcaceae; g__Succiniclasticum; s__Succiniclasticum_ruminis
OTU70	18	12	k__Bacteria; p__Firmicutes; c__Bacilli; Unclassified; Unclassified; Unclassified; Unclassified
OTU71	7	7	k__Bacteria; p__Firmicutes; c__Clostridia; Unclassified; Unclassified; Unclassified; Unclassified
OTU72	9	12	k__Bacteria; p__Firmicutes; c__Clostridia; Unclassified; Unclassified; Unclassified; Unclassified
OTU73	40	34	k__Bacteria; p__Firmicutes; c__Bacilli; Unclassified; Unclassified; Unclassified; Unclassified
OTU74	9	10	k__Bacteria; p__Firmicutes; c__Bacilli; Unclassified; Unclassified; Unclassified; Unclassified
OTU75	8	12	k__Bacteria; p__Planctomycetota; c__Planctomycetes; o__Pirellulales; f__Pirellulaceae; Unclassified; Unclassified
OTU76	37	57	k__Bacteria; p__Bacteroidota; c__Bacteroidia; o__Bacteroidales; f__Prevotellaceae; g__Prevotella; s__Prevotella_sp
OTU77	10	18	k__Bacteria; p__Bacteroidota; c__Bacteroidia; o__Bacteroidales; f__Prevotellaceae; g__Prevotella; s__Prevotella_sp
OTU78	2	6	k__Bacteria; p__Firmicutes; c__Bacilli; Unclassified; Unclassified; Unclassified; Unclassified
OTU79	7	6	k__Bacteria; p__Cyanobacteria; Unclassified; Unclassified; Unclassified; Unclassified; Unclassified
OTU8	66	93	k__Bacteria; p__Bacteroidota; c__Bacteroidia; o__Bacteroidales; f__Prevotellaceae; g__Prevotella; Unclassified
OTU80	22	30	k__Bacteria; p__Bacteroidetes; c__Bacteroidia; o__Bacteroidales; f__Prevotellaceae; Unclassified; Unclassified
OTU81	30	72	k__Bacteria; p__Planctomycetota; c__Planctomycetes; o__Pirellulales; f__Pirellulaceae; Unclassified; Unclassified
OTU82	45	26	k__Bacteria; p__Firmicutes; c__Clostridia; o__Oscillospirales; f__Oscillospiraceae; Unclassified; Unclassified
OTU83	17	18	k__Bacteria; p__Firmicutes; c__Bacilli; Unclassified; Unclassified; Unclassified; Unclassified
OTU84	20	18	k__Bacteria; p__Verrucomicrobiota; c__Verrucomicrobiae; Unclassified; Unclassified; Unclassified; Unclassified
OTU85	2	3	k__Bacteria; p__Patescibacteria; c__Saccharimonadia;

			o__Saccharimonadales; f__Saccharimonadaceae; g__Candidatus_Saccharimonas; s__Candidatus_Saccharimonas_aalborgensis
OTU86	18	23	k__Bacteria; p__Planctomycetota; c__Planctomycetes; o__Pirellulales; f__Pirellulaceae; Unclassified; Unclassified
OTU87	3	17	k__Bacteria; p__Firmicutes; c__Clostridia; Unclassified; Unclassified; Unclassified; Unclassified
OTU88	12	7	k__Bacteria; p__Spirochaetota; c__Spirochaetia; o__Spirochaetales; f__Spirochaetaceae; g__Treponema; s__Treponema_saccharophilum
OTU89	23	16	k__Bacteria; p__Bacteroidota; c__Bacteroidia; o__Bacteroidales; f__Rikenellaceae; g__Bact; s__Bacteroidales_bacterium
OTU9	43	17	k__Bacteria; p__Firmicutes; c__Clostridia; o__Lachnospirales; f__Lachnospiraceae; Unclassified; Unclassified
OTU90	21	11	k__Bacteria; p__Firmicutes; c__Bacilli; Unclassified; Unclassified; Unclassified; Unclassified
OTU91	33	30	k__Bacteria; p__Firmicutes; c__Bacilli; Unclassified; Unclassified; Unclassified; Unclassified
OTU92	53	61	k__Bacteria; p__Bacteroidota; c__Bacteroidia; o__Bacteroidales; f__Prevotellaceae; g__Prevotella; s__Prevotella_sp
OTU93	41	22	k__Bacteria; p__Firmicutes; c__Clostridia; Unclassified; Unclassified; Unclassified; Unclassified
OTU94	16	19	k__Bacteria; p__Firmicutes; c__Clostridia; o__Lachnospirales; f__Lachnospiraceae; g__Lachnobacterium; s__Lachnobacterium_bovis
OTU95	27	22	k__Bacteria; p__Bacteroidota; c__Bacteroidia; o__Bacteroidales; f__Prevotellaceae; g__Prevotella; s__Prevotella_sp
OTU96	3	7	k__Bacteria; p__Firmicutes; c__Bacilli; Unclassified; Unclassified; Unclassified; Unclassified
OTU97	35	18	k__Bacteria; p__Firmicutes; c__Bacilli; Unclassified; Unclassified; Unclassified; Unclassified
OTU98	13	17	k__Bacteria; p__Firmicutes; c__Clostridia; Unclassified; Unclassified; Unclassified; Unclassified
OTU99	5	0	k__Bacteria; p__Firmicutes; c__Clostridia; Unclassified; Unclassified; Unclassified; Unclassified
