# Supplementary material for: Fermented Soybean Meal Replacement in the Diet of Lactating Holstein Dairy Cows: Modulated Rumen Fermentation and Ruminal Microflora
Source: Front Microbiol. 2021 Jan 29;12:625857. doi: 10.3389/fmicb.2021.625857 (PMC7879537; doi:10.3389/fmicb.2021.625857)
Supplement: Supplementary file 7 [file Table_3.pdf]

Supplementary Material

Fermented soybean meal replacement in the diet of lactating Holstein dairy cows: improved rumen fermentation and modulated ruminal microflora

Zuo Wang [1], Yuannian Yu [1], Xinyao Li [1], Hongyan Xiao [1], Peihua Zhang [1], Weijun Shen [1], Fachun Wan [1], Jianhua He [1], Shaoxun Tang [2]*, Zhiliang Tan [2], Duanqin Wu [3]*, and Hui Yao [4]

[1] College of Animal Science and Technology, Hunan Agricultural University, Changsha, Hunan 410128, China

[2] CAS Key Laboratory of Agro-Ecological Processes in Subtropical Region, National Engineering Laboratory for Pollution Control and Waste Utilization in Livestock and Poultry Production, Hunan Provincial Key Laboratory of Animal Nutrition & Physiology and Metabolism, Institute of Subtropical Agriculture, Chinese Academy of Sciences, Changsha, Hunan 410125, China

[3] Institute of Bast Fiber Crops, Chinese Academy of Agricultural Sciences, Changsha, Hunan, 410205, China

[4] Nanshan Dairy Co. Ltd., Shaoyang, Hunan 422500, China

*** Correspondence:**

Shaoxun Tang; Duanqin Wu

shaoxuntang@163.com; wuduanqin@caas.cn

Supplementary Tables

Table S3. Comparison of the assigned KEGG pathways between the SBM and FSBM treatments using t-test based on Tax4Fun

Class1	Class2	FSM: mean (%)	FSM: std (%)	SM: mean (%)	SM: std. (%)	p-values (corrected)
Metabolism	Carbohydrate metabolism	15.58704	0.268112	15.58526	0.273845	0.987846
Metabolism	Amino acid metabolism	10.87998	0.359651	11.04327	0.588136	0.44222
Environmental Information Processing	Membrane transport	7.748967	0.532377	7.636847	0.51211	0.619696
Metabolism	Metabolism of cofactors and vitamins	7.544012	0.269322	7.602097	0.46445	0.723984
Metabolism	Nucleotide metabolism	7.342865	0.204104	7.333148	0.332878	0.935122
Metabolism	Energy metabolism	6.781376	0.089131	6.830054	0.173186	0.41904
Genetic Information Processing	Translation	6.680242	0.207989	6.635727	0.192379	0.607533
Genetic Information Processing	Replication and repair	6.403492	0.185931	6.331396	0.194984	0.384427
Environmental Information Processing	Signal transduction	5.779532	0.443632	5.814364	0.501135	0.86456
Metabolism	Glycan biosynthesis and metabolism	4.386194	0.263573	4.377293	0.258102	0.93694
Genetic Information Processing	Folding, sorting and degradation	3.029204	0.09859	3.031511	0.097735	0.956538
Metabolism	Lipid metabolism	2.630351	0.054324	2.607504	0.14024	0.622085
Metabolism	Metabolism of terpenoids and polyketides	2.184379	0.068996	2.180438	0.074484	0.898742
Metabolism	Metabolism of other amino acids	2.148208	0.034299	2.134286	0.082947	0.614618
Metabolism	Xenobiotics biodegradation and metabolism	2.144307	0.098016	2.158314	0.090079	0.730461

Cellular Processes	Cell growth and death	1.855741	0.0186	1.856289	0.041019	0.968352
Cellular Processes	Cell motility	1.557721	0.220617	1.521795	0.241917	0.719411
Human Diseases	Infectious diseases: Bacterial	1.47835	0.039521	1.470953	0.072037	0.768863
Metabolism	Biosynthesis of other secondary metabolites	0.839091	0.023841	0.842089	0.032793	0.808751
Organismal Systems	Digestive system	0.551422	0.059923	0.556007	0.056976	0.855781
Organismal Systems	Endocrine system	0.435036	0.02084	0.429311	0.03388	0.638786
Cellular Processes	Transport and catabolism	0.374924	0.026106	0.386307	0.043343	0.465172
Genetic Information Processing	Transcription	0.295334	0.010839	0.28925	0.016315	0.315792
Organismal Systems	Environmental adaptation	0.251061	0.010754	0.250116	0.023706	0.905728
Metabolism	Global and overview maps	0.174214	0.004075	0.175563	0.009874	0.681257
Organismal Systems	Immune system	0.143798	0.011364	0.14494	0.011352	0.815787
Human Diseases	Neurodegenerative disease	0.138299	0.007423	0.13813	0.010651	0.966145
Organismal Systems	Nervous system	0.134678	0.00257	0.137175	0.002621	0.034331
Human Diseases	Infectious diseases: Parasitic	0.128946	0.009329	0.127479	0.008805	0.70806
Human Diseases	Drug resistance: Antimicrobial	0.084519	0.015708	0.084758	0.0178	0.973721
Human Diseases	Endocrine and metabolic diseases	0.068314	0.002094	0.068606	0.003353	0.80865
Human Diseases	Cancers: Overview	0.0661	0.001664	0.067065	0.002674	0.322706
Human Diseases	Immune diseases	0.05831	0.003551	0.056773	0.004408	0.377955
Human Diseases	Cancers: Specific types	0.044896	0.00249	0.045675	0.004108	0.597434
Organismal Systems	Excretory system	0.028545	0.0017	0.028776	0.002269	0.789594
Organismal Systems	Circulatory system	0.006457	0.000624	0.006526	0.000687	0.807013
Human Diseases	Infectious diseases: Viral	0.004503	0.000712	0.004781	0.001584	0.60269

Human Diseases	Substance dependence	0.003829	0.000756	0.003872	0.001266	0.924177
Human Diseases	Neurodegenerative diseases	0.003096	0.000644	0.003452	0.001108	0.368911
Human Diseases	Cardiovascular diseases	0.001635	0.000179	0.001749	0.000281	0.2693
Environmental Information Processing	Signaling molecules and interaction	0.000487	0.000151	0.000482	0.00015	0.948155
Cellular Processes	Cellular community - eukaryotes	0.000486	0.000151	0.000479	0.000149	0.914691
Human Diseases	Infectious disease: viral	5.46E-05	2.6E-05	8.98E-05	5.9E-05	0.090057
